# Generalized nonparametric temporal modeling of recurrent events with application to a malaria vaccine trial

**DOI:** 10.1093/biomtc/ujaf146

**Published:** 2025-11-21

**Authors:** Fei Heng, Yanqing Sun, Jing Xu, Peter B Gilbert

**Affiliations:** Department of Mathematics and Statistics, University of North Florida, Jacksonville, FL 32224, United States; Department of Mathematics and Statistics, University of North Carolina at Charlotte, Charlotte, NC 28223, United States; Vaccine and Infectious Disease, Fred Hutchinson Cancer Center, Seattle, WA 98109, United States; Vaccine and Infectious Disease, Fred Hutchinson Cancer Center, Seattle, WA 98109, United States; Department of Biostatistics, University of Washington, Seattle, WA 98109, United States

**Keywords:** double kernel smoothing, intensity model, local linear smoothing, nonparametric maximum likelihood estimation, recurrent events, temporal effects

## Abstract

Motivated by a malaria vaccine efficacy trial, this paper investigates generalized nonparametric temporal models of intensity processes with multiple time scales. Through the choice of link functions, the proposed models encompass a wide range of models such as the multiplicative temporal intensity model and the additive temporal intensity model. A maximum likelihood estimation procedure is developed to estimate the effects of two time-scales via the local linear smoothing with double kernels. Computational algorithms are developed to facilitate applications of the proposed method. An adaptive algorithm is developed to overcome the challenges of overlapping covariates. A cross-validation bandwidth selection procedure based on the logarithm of likelihood criteria is discussed. The asymptotic properties of the proposed estimators are investigated. Our simulation study shows that the proposed methods have satisfactory finite sample performance for both the multiplicative temporal intensity model and additive temporal intensity model. The proposed methods are applied to analyze the MAL-094/MAL-095 malaria vaccine efficacy trial data to investigate how the new malaria infection risk changes over time and how a prior infection or vaccination changes the future infection risk. The proposed method provides new insight into the protective effects of the malaria vaccine against new malaria infections and how the vaccine efficacy is modified by the history of prior malaria infection over time.

## 1 INTRODUCTION

Malaria is a life-threatening disease caused by *Plasmodium falciparum* parasites that can transmit from human to human through the bite of an infected Anopheles mosquito. There were an estimated 249 million malaria cases and 608 000 malaria deaths globally in 2022. African region was home to 94% of malaria cases and 95% of malaria deaths. Children under the age of 5 are disproportionately affected by malaria with about 80% of all malaria deaths (WHO, 2023). A person can be infected with malaria multiple times with different (and possibly the same) malaria parasite strains. MAL-094 (NCT03276962, ClinicalTrials.gov) was a vaccine efficacy trial of four different malaria vaccines (RTS,S/AS01$_{\rm E}$) versus a rabies vaccination control arm conducted by GSK and the PATH Malaria Vaccine Initiative in children 5–17 months of age living in sub-Saharan Africa (RTS,S Clinical Trials Partnership, 2012; Samuels et al., 2022). The MAL-094 trial participants received rabies vaccine or one of four versions of the malaria vaccine in different doses and schedules (Month 0, 1, 2, and between 1 and 3 boosts at different visits through Month 38). The MAL-095 sub-study of MAL-094 (NCT03281291, ClinicalTrials.gov) conducted frequent diagnostic testing for malaria infection and provides an opportunity to understand the protective effects of the malaria vaccines and the effects of a prior malaria infection on the risk of future malaria infections.

Motivated by the malaria vaccine trial, we develop nonparametric temporal intensity models to assess the risk factors of event occurrences, and how the occurrences of events are affected by concomitant variables, interventions, and event history. Modeling of recurrent events can be based on counting processes that register occurrences of events. The intensity of a counting process describes the instantaneous probability of an event occurrence over time conditional on the history, which is commonly used to study event history; see Andersen et al. (1993).

When the intensity of a counting process is only a function of time not depending on the event history, the counting process is said to be of Poisson-type for which the number of events in nonoverlapping time intervals are independent. The recurrent event processes with constant intensity are known as homogeneous Poisson processes, otherwise they are called inhomogeneous Poisson processes. If the intensity depends on the event history in a way such that it is a function of backward recurrence time, ie, time elapsed since the occurrence of the last event, then the counting process is a renewal process, and in this case the successive gap times between the events are independent and identically distributed. It is well known that homogeneous Poisson processes are also renewal processes; the families of Poisson-type processes and renewal processes are distinct otherwise (Ross, 1995).

There is an extensive statistical literature on modeling the intensity of the Poisson-type counting processes. Aalen (1978) investigated a family of multiplicative intensity models for counting processes. Andersen and Gill (1982) studied the Cox model for the intensity of counting processes. Wang et al. (2001) developed statistical methods and theory for modeling recurrent events with informative censoring under a multiplicative intensity model. Zeng and Lin (2006) studied a class of semiparametric transformation models for counting processes. Chen et al. (2013) studied the Cox-type intensity model for overdispersed recurrent event data allowing the covariate effects to depend on the time since treatment switching. Scheike (2001) investigated a general additive regression model for counting processes where the time-varying effects depend on two time-scales. A comprehensive review of general intensity-based models is given by Cook and Lawless (2007).

The Poisson-type intensity models assume that the intensity of event occurrence for a participant that just experienced an event occurrence is identical to the intensity just prior to the event occurrence. In the malaria disease situation and for many other infectious diseases; however, a prior infection may stimulate immune responses and hence change the future infection risk. Thus the Poisson-type models have some limitations for applications similar to the malaria case. On the other hand, the models for renewal processes also present challenges since the gap times between consecutive malaria infections may change in distribution. More flexible models that can model both Poisson and renewal-type behavior of recurrent events are desirable; see the many examples discussed in Peña and Hollander (2004). Lawless and Thiagarajah (1996) modeled time trends and effects of past events through the parametric Cox-type intensity models. Peña and Hollander (2004) and Peña et al. (2007) proposed a class of semiparametric intensity models that incorporates the effects of covariates, the impact of event counts, and the effect of the backward recurrence time (the time elapsed since the last event), each of which contributes multiplicatively to the intensity. Asymptotic properties of these semiparametric estimators were established by Peña (2016). Despite the important progresses made in the dynamic modeling of recurrent events, challenges remain. Most existing literature is based on the Cox-type intensity models in that the covariate effects are constant, presenting some disadvantages for the malaria application since vaccine efficacy against clinical malaria disease has been observed to wane over time (White et al., 2015).

In this paper, we investigate generalized nonparametric temporal models for the intensity of event occurrences where the covariate effects are functions of time or time-varying event history. The proposed models have features of the generalized semiparametric mixed varying-coefficients models of Sun et al. (2019). But the two models focus on different types of data. While Sun et al. (2019) considered the conditional mean model for the longitudinal response, the proposed models are for the intensities of the recurrent events. The new models combine features of inhomogeneous Poisson models and renewal-type models. This dual perspective allows for a comprehensive understanding of temporal dynamics. The renewal component of the proposed temporal intensity models can be used to address whether and how occurrence of a prior event changes the likelihood of a future event. The (calendar) time-varying component is equipped to accommodate the time-varying nature of the event intensity, which is particularly relevant for infectious diseases. The proposed models allow the covariate effects to vary with calendar time and effective time, a transformed time scale that captures the underlying process dynamic—such as time-varying exposure and backward recurrence time. Through the choice of link functions, the proposed models encompass a wide range of models such as the multiplicative temporal intensity model and the additive temporal intensity model. To the best of our knowledge, these models have not been studied for recurrent events. We develop a maximum likelihood estimation procedure to estimate the effects of two time scales using the local linear smoothing method with double kernels. Computational algorithms are developed to facilitate applications of the proposed methods. An adaptive algorithm is developed to overcome the challenges of overlapping covariates. We consider a $K$-fold cross-validation bandwidth selection procedure based on the likelihood criteria. The asymptotic properties of the proposed estimators, including the uniform consistency and weak convergence, are investigated. The proofs of the asymptotic results are challenging because of two dimensional kernel smoothing and that the covariate effects intertwine across two time scales. The performance of the proposed methods is demonstrated through extensive simulations. The methods are applied to the malaria vaccine trial MAL-094 to provide new insight into the protective effects of the malaria vaccine against new malaria infections and how the vaccine efficacy is modified by the history of prior malaria infection.

The structure of this paper is organized as follows. In Section 2, we introduce the generalized nonparametric temporal intensity models and present the maximum likelihood estimation procedure via double kernel smoothing. We also investigate the temporal effects in two time scales—the calendar time and effective time and discuss selections of kernel functions and bandwidths. The asymptotic properties of the proposed estimators are established in Section 3. The results of the simulation studies are shown in Section 4. Section 5 presents an application. Some concluding remarks are given in Section 6. Additional information is available in Supporting Information at the Biometrics website.

## 2 NONPARAMETRIC TEMPORAL INTENSITY MODELS AND ESTIMATION

### 2.1 Generalized nonparametric temporal intensity models

Suppose that there is a random sample of $n$ participants. For participant $i$, the recurrent events such as malaria infections occur at times $0\le T_{i,1}< T_{i,2} <\cdots$. Modeling of the recurrent events can be based on the counting process ${N_i^{*}(t) = \sum _{j=1}^{\infty } I(T_{i,j} \le t)}$, which registers the number of events for the $i$th participant by time $t$, where $I(\cdot )$ is the indicator function. Let $\lbrace \mathcal {F}_{it}\rbrace$ denote the event and covariate history up to time $t$ for participant $i$. Let $\Delta N_i^{*}(t) = N_i^{*}(t) - N_i^{*}(t^-)$, where $N_i^{*}(t^-)$ is defined as the left limit of $N_i^{*}(\cdot )$ at $t$. Then $\Delta N_i^{*}(t) =1$ at the jump times and 0 otherwise. The intensity of a counting process is defined by $\lambda _i(t)=\lim _{\Delta t\downarrow 0} {\Pr (N_i^{*}(t+\Delta t^-)-N_i^{*}(t^-)=1|\mathcal {F}_{{it}^-})}/{\Delta t}$, thus $\lambda _i(t)dt$ is the instantaneous probability of an event occurring in $[t,t+dt)$ conditional on the history $\mathcal {F}_{{it}^-}$. Let $Y_i(t)$ be a $\lbrace 0,1\rbrace$-valued censoring process for participant $i$. The observed event process can be expressed as $N_i(t)=\int _0^tY_i(s)dN_i^{*}(s)$. In the right censoring scenario, $Y_i(t)=I(C_i\ge t)$ and $N_i(t)=N^{*}(t \wedge C_i),$ where $C_i$ is the end of follow-up time $\tau$ or censoring time whichever comes first. Suppose that $U_{i}(t)$ is the effective time such as time-varying exposure and backward recurrence time at time $t$. Let $X_i(t)$ be the $p_1$ dimensional time-dependent covariates whose effects vary over calendar time, and $Z_i(t)$ the $p_2$ dimensional time-dependent covariates whose effects are functions of $U_i(t)$. Assume that $\lbrace N_i(\cdot ), X_i(\cdot ), Z_i(\cdot ), U_i(\cdot ), i=1,\cdots ,n\rbrace$ are independent identically distributed random processes. Let $\mathcal {F}_{t}= \vee _{i=1}^n \lbrace \mathcal {F}_{it}\rbrace$, $0\le t\le \tau$, be the filtration for all participants. Let $\mathcal {G}_{t}$, $0\le t\le \tau$, be the filtration generated by the observed event history, covariate processes and censoring for all participants. The censoring is assumed to be independent in the sense that $E(dN_i(t)|\mathcal {G}_{t^-})=E(dN_i(t)|\mathcal {F}_{t^-})=\lambda _i(t)Y_i(t)dt$ for $0\le t\le \tau$ (Andersen et al., 1993).

The proposed generalized nonparametric temporal intensity model postulates that


(1) \begin{eqnarray*}
\lambda _i(t)=g^{-1}\lbrace \alpha ^{ T}(t)X_i(t)+\gamma ^T(U_{i}(t))Z_i(t)\rbrace ,
\end{eqnarray*}

for $0\le t\le \tau$, where $\alpha (\cdot )$ is a $p_1$-dimensional vector of unspecified functions, $\gamma (\cdot )$ is a $p_2$-dimensional vector of unspecified functions, and $g(\cdot )$ is a known link function such as the identity function or logarithm function. The identity link function yields an additive intensity model while the logarithm link gives a multiplicative intensity model. Setting the first component of ${X_{i}}(t)$ as 1 provides the nonparametric baseline function. Model (1) allows for flexibility in capturing complex temporal patterns. Specifically, $\alpha (t)$ represents the temporal effect of the covariate $X_i(t)$ at calendar time $t$, while $\gamma (u)$ denotes the temporal effect of $Z_i(t)$ evaluated at effective time $u$.

**FIGURE 1:**
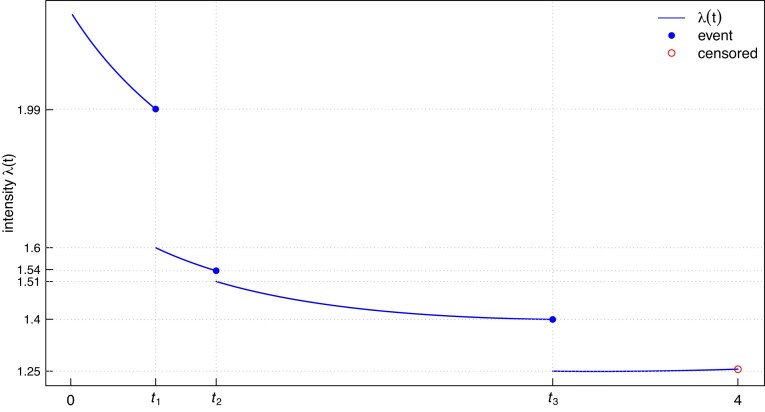
A simulated sample path of the intensity $\lambda _i(t)=\exp \lbrace {\alpha _0}(t)+{\gamma }(t-T_{N_i(t^-)})I(N_i(t^-)>0)W_{i}\rbrace$, where $\alpha _0(t)=1-\log (1.2+0.4\log (1+t))$ and $\gamma (u)=-0.4+0.1u$, $W_i$ is a uniform random variable on [0,1], for $0\le t\le 4$. The censoring time is $C_i=\min (\tau , C_i^{*})$ where $C_i^{*}$ has uniform distribution on (3,8). The figure is for $\lambda _i(t)$ with $W_i=0.5455$, where $t_k$, $k=1,2,\ldots$, are observed event times.

With the specification $U_{i}(t)=t-T_{i,{N_{i}(t^-)}}$ and $Z_i(t)=W_i(t)I(N_i(t^-)>0)$, model (1) clearly delineates covariate effects along both calendar and backward recurrence time scales. This setup resembles a simplified Hawkes-type self-exciting process, where the intensity depends on the most recent past event rather than the full event history (Hawkes, 1971). A notable special case is obtained by letting $X_i(t)=W_i(t)=1$ and $Z_i(t)=I(N_i(t^-)>0)$, reducing model (1) to: $\lambda _i(t)=g^{-1}\big \lbrace \alpha (t)+\gamma (t-T_{i,{N_{i}(t^-)}}) I(N_i(t^-)>0)\big \rbrace$. Figure 1 illustrates this submodel under a log-link function. The sample path shows that the intensity process is left-continuous and jumps immediately following each event, highlighting the impact of recent infection history on future risk.

Another example sets $U_i(t)=t-V_i$ and $Z_{i}(t)=I(t>V_i)$ where, for instance, $V_i$ represents the time of vaccination or intervention. In this case, $\gamma (u)$ captures how the event intensity evolves with time since the intervention, highlighting the post-intervention effect at effective time $u$. More examples can be found in Peña and Hollander (2004). While both model (1) and the model of Peña and Hollander (2004) aim to account for event history in modeling the intensity and share overlapping submodels, model (1) does not encompass the Peña–Hollander model. Instead, it offers greater flexibility by allowing for more general temporal effects.

### 2.2 Nonparametric maximum likelihood estimation via double kernels

In the following, we propose a maximum likelihood estimation procedure for model (1) via local linear smoothing with double kernels (Fan and Gijbels, 1996; Qi et al., 2017). Let ${\cal U}$ be the support of $U_i(t)$. First, we assume that $X_i(t)$ and $Z_i(t)$ do not have common covariates. The scenario where $X_i(t)$ and $Z_i(t)$ have common components will be dealt with next. Assume that $\alpha (t)$ for $t\in [0,\tau ]$ and $\gamma (u)$ for $u\in {\cal U}$ are smooth functions that are first and second order differentiable. Let $\dot{\alpha }(\cdot )$ and $\dot{\gamma }(\cdot )$ be the derivatives of $\alpha (\cdot )$ and $\gamma (\cdot )$, respectively. Let ${\cal U}^o$ be an open subset of ${\cal U}$. For each $t_0\in (0,\tau )$ and $u_0\in {\cal U}^o$, let ${\cal N}_{t_0}$ be a neighborhood of $t_0$ and ${\cal N}_{u_0}$ a neighborhood of $u_0$. By the first order Taylor approximation, $\alpha (t)=\alpha (t_0)+\dot{\alpha }(t_0)(t-t_0)+O((t-t_0)^2)$ for $t \in {\cal N}_{t_0}$ and $\gamma (u)=\gamma (u_0)+\dot{\gamma }(u_0)(u-u_0)+O((u-u_0)^2)$ for $u\in {\cal N}_{u_0}$. For $t \in {\cal N}_{t_0}$ and $U_i(t) \in {\cal N}_{u_0}$, the intensity $\lambda _i(t)$ can be approximated by $\lambda ^{*}_i(t,\vartheta ^{*}| t_0,u_0) =\varphi \lbrace \vartheta ^{*T}(t_0,u_0)\tilde{X}_i^{*}(t,t_0,u_0)\rbrace ,$ where $\varphi (\cdot )=g^{-1}(\cdot )$, $\vartheta ^{*}(t_0,u_0)=(\alpha ^T(t_0),\gamma ^T(u_0),\dot{\alpha }^T(t_0),\dot{\gamma }^T(u_0))^T$, $\tilde{X}_i^{*}(t| t_0,u_0)=({X}^T_{i}(t),$  $Z_{i}^T(t),{X}^T_{i}(t) (t-t_0),Z_{i}^T(t)(U_i(t)-u_0))^T$.

At each $t_0$ and $u_0$, let $K_{h,b}(t,U_i(t)| t_0,u_0)=K_h(t-t_0)K_b(U_i(t)-u_0)$ be the bivariate kernel, where $K_h(\cdot )=K_1(\cdot /h)/h$ and $K_b(\cdot )=K_2(\cdot /b)/b$. Here, $K_1(\cdot )$ and $K_2(\cdot )$ are kernel functions, and $h=h_n$ and $b=b_n$ are bandwidth parameters that depend on $n$. By construction of the likelihood of counting processes (Daley and Vere-Jones, 2003) and applying the local smoothing method, we obtain the local log-likelihood function for $\alpha (\cdot )$ and $\gamma (\cdot )$ at $(t_0,u_0)$:


(2) \begin{eqnarray*}
\ell _\vartheta (\vartheta ^{*}| t_0,u_0) &=&\sum _{i=1}^n \int _0^\tau K_{h,b}(t,U_i(t)| t_0,u_0) \\
&&\times\,\lbrace \log (\lambda ^{*}_i(t,\vartheta ^{*}| t_0,u_0))\, dN_i(t) \\
&&-\, Y_i(t)\lambda ^{*}_i(t,\vartheta ^{*}| t_0,u_0)\, dt \rbrace .
\end{eqnarray*}

A detailed derivation of (2) is provided in Web Appendix B. By taking the derivative of the local log-likelihood function with respect to $\vartheta ^{*}$, we have the local score function:


(3) \begin{eqnarray*}
U(\vartheta ^{*}|t_0,u_0)&=&\sum _{i=1}^n \int _0^\tau K_{h,b}(t,U_i(t)| t_0,u_0)
\frac{\dot{\lambda _i^{*}}(t, \vartheta ^{*}|t_0,u_0)}{ \lambda _i^{*}(t, \vartheta ^{*}|t_0,u_0)}\\
&&\times\, \lbrace dN_i(t) -Y_i(t) \lambda _i^{*}(t, \vartheta ^{*}|t_0,u_0)dt \rbrace\\
&&\times\, \widetilde{X}^{*}_i(t|t_0,u_0),
\end{eqnarray*}

where $\dot{\lambda _i^{*}}(t, \vartheta ^{*}|t_0,u_0)=\dot{\varphi }\big \lbrace \vartheta ^{*{ \mathrm{\scriptscriptstyle T} }}(t_0,u_0)\widetilde{X}_i^{*}(t|t_0,u_0)\big \rbrace$ and $\dot{\varphi }(\cdot )$ is the first derivative of ${\varphi }(\cdot )$ with respect to $\vartheta ^{*}$. The bivariate estimator $\hat{\vartheta }^{*}(t_0,u_0)$ can be obtained by solving $U(\vartheta ^{*}|t_0,u_0)=0$ through the Newton–Raphson method.

Let $\hat{\alpha }(t_0,u_0)$ include the first $p_1$ elements of $\hat{\vartheta }^{*}(t_0,u_0)$ corresponding to the position of $\alpha (t_0)$ in $\vartheta ^{*}(t_0,u_0)$. Let $\hat{\gamma }(t_0,u_0)$ include the elements of $\hat{\vartheta }^{*}(t_0,u_0)$ corresponding to the position of $\gamma (u_0)$ in $\vartheta ^{*}(t_0,u_0)$. Then, $\hat{\vartheta }(t_0,u_0)=(\hat{\alpha }^{{ \mathrm{\scriptscriptstyle T} }}(t_0,u_0), \hat{\gamma }^{{ \mathrm{\scriptscriptstyle T} }}(t_0,u_0))^{{ \mathrm{\scriptscriptstyle T} }}$ is an estimator of $(\alpha (t_0),\gamma (u_0))$. However, the estimator $\hat{\alpha }(t_0,u_0)$ of $\alpha (t_0)$ is inefficient because it only utilizes the local observations with $U_i(t) \in {\cal N}_{u_0}$ for $t \in {\cal N}_{t_0}$. Similarly, the estimator $\hat{\gamma }(t_0,u_0)$ of $\gamma (u_0)$ only utilizes the local observations for $t \in {\cal N}_{t_0}$ with $U_i(t)=u_0$. More efficient estimators for $\alpha ^{{ \mathrm{\scriptscriptstyle T} }}(t_0)$ and $\gamma ^{{ \mathrm{\scriptscriptstyle T} }}(u_0)$ can be achieved by aggregating the estimated bivariate functions $\hat{\alpha }(t_0,u_0)$ and $\hat{\gamma }(t_0,u_0)$ along each direction:


(4) \begin{eqnarray*}
&& \hat{\alpha }(t_0)= n^{-1}\sum _{i=1}^{n} \hat{\alpha }(t_0,U_i(t_0)), \\
&& \hat{\gamma }(u_0)= n_{u_0}^{-1}\sum _{t_{u_0}\in {\cal V}_{u_0}} \hat{\gamma }(t_{u_0},u_0),
\end{eqnarray*}

where ${\cal V}_{u_0}=\cup _{i=1}^n U_i^{-1}(u_0)$, $U_i^{-1}(u_0)=\lbrace t: U_i(t)=u_0\rbrace$, and $n_{u_0}=|{\cal V}_{u_0}|$ is the cardinality of ${\cal V}_{u_0}$.

The idea of using aggregation to obtain more efficient estimators is well justified. Theorems 1 and 2 of Section 3 show that the asymptotic variances of $\hat{\alpha }(t)$ and $\hat{\gamma }(u)$ are of order $1/(nh)$ and $1/(nb)$, respectively. In contrast, Lemma 2 in Web Appendix A shows that the asymptotic variances of the unaggregated estimators are of order $1/(nhb)$. Therefore, the aggregated estimators $\hat{\alpha }(t)$ and $\hat{\gamma }(u)$ are more efficient than their unaggregated counterparts for $h,b\rightarrow 0$ as $n\rightarrow \infty$.

### 2.3 Estimation of the temporal model with two time-scales

In this section, we focus on the temporal intensity model (1) with two time-scales by letting $U_{i}(t)=t-T_{i,{N_{i}(t^-)}}$ and $Z_i(t)=W_i(t)I(N_i(t^-)>0)$. In particular, we investigate the intensity model (1) with the temporal effects in two time-scales:


(5) \begin{eqnarray*}
\lambda _i(t)&=&g^{-1}\lbrace \alpha ^{ T}(t)X_i(t)\\
&&+\,\gamma ^T(t-T_{i,{N_{i}(t^-)}})W_i(t)I(N_i(t^-)>0) \rbrace ,
\end{eqnarray*}

for $0\le t\le \tau$, where $\alpha (t)$ is the (calendar) time-varying effects of covariate $X_i(t)$, and $\gamma (u)$ is the (backward recurrence) time-varying effects of covariate $W_i(t)$ that varies with $u$, the time elapsed since the last event.

If $X_i(t)$ and $W_i(t)$ do not have common covariates, the estimation procedure developed in Section 2.2 can be used to estimate $\alpha (t)$ and $\gamma (u)$. However, when covariates $X_i(t)$ and $W_i(t)$ have overlapping components, additional work is needed to estimate $\alpha (t)$ and $\gamma (u)$. To exemplify, let us consider an important special model $\lambda _i(t)=g^{-1}\lbrace \alpha (t)+\gamma (U_i(t))I(N_i(t^-)>0)\rbrace$ with $X_i(t)=W_i(t)=1$, where $U_{i}(t)=t-T_{i,{N_{i}(t^-)}}$. Under local linear smoothing, the expression $\vartheta ^{*{ \mathrm{\scriptscriptstyle T} }}(t_0,u_0)\widetilde{X}_i^{*}(t|t_0,u_0)$ within the estimating equation (3) becomes $\alpha (t_0)+\gamma (u_0)I(N_i(t^-)>0)+\dot{\alpha }(t_0)(t-t_0)+\dot{\gamma }(u_0)(U_i(t)-u_0)) I(N_i(t^-)>0).$ Hence, $\alpha (t_0)$ and $\gamma (u_0)$ cannot be identified locally if $P(N_i (t^-)>0)=1$ for $t \in {\cal N}_h(t_0)$, where ${\cal N}_h(t_0)=(t_0-h,t_0+h)$. On the other hand, $\gamma (u_0)$ cannot be estimated if $P(N_i (t^-)>0)=0$ for $t \in {\cal N}_h(t_0)$.

To avoid the difficulty in solving the equation $U(\vartheta ^{*}|t,u)=0$, we propose an adaptive algorithm to estimate $\alpha (t)$ and $\gamma (u)$ based on the data observed in a neighborhood of $s$, for $s\le t$. Based on the estimates $\hat{\alpha }(t_0)$ and $ \hat{\gamma }(u_0)$ that are available for $(t_0,u_0)$ using the aggregated double kernel estimator (4) and using the equation $\alpha (t)=\alpha (t_0)+\int _{t_0}^t \dot{\alpha }(s)\, ds$, we estimate $\alpha (t)$ adaptively based on the estimated $\alpha (s)$ and its derivative $\dot{\alpha }(s)$ for $s\le t$. To this end, we assume that $0<P(N_i(t^-)=0)< 1$ and $P(U_i(t) \in {\cal N}_b(u_0))>0$ holds for $t \in {\cal N}_h(t_0)$, where ${\cal N}_b(u_0)=(u_0-b,u_0+b)$. The assumption ensures the extended covariate matrix composed of $\widetilde{X}_i^{*}(t|t_0,u_0)$, $i=1,\ldots ,n$, maintains full rank. The condition $0<P(N_i(t-)=0)< 1$ for $t \in {\cal N}_h(t_0)$, which holds true at the beginning of the study, eg, $t$ near 0, ensures that $\alpha (t_0)$ and $\gamma (u_0)$ are locally identifiable, while $P(U_i(t) \in {\cal N}_b(u_0))>0$ for $t \in {\cal N}_h(t_0)$ is the condition needed so that the kernel weight $K_b(U_i(t)-u_0)$ does not equal to zero with probability 1.

*Adaptive Estimation Algorithm:*

Let $\lbrace t_k,\, k=1,\ldots , K\rbrace$ be the equally spaced grid points of the increment $\Delta t$ over $(0,\tau )$ and $\lbrace u_j,\, j=1,\ldots , J\rbrace$ be the grid points for $u\in {\cal U}^o$. For ease of notation, we let $t_0=t_k$ and $u_0=u_j$ be on the grid points of $(0,\tau )$ and ${\cal U}^o$, respectively.

Step 1. We estimate $\vartheta ^{*}(t_0,u_0)$ by solving $U(\vartheta ^{*}|t_0,u_0)=0$ for $(t_0,u_0) \in \Delta =\lbrace 0\le u \le t \le t^{*}\rbrace$, where $t^{*}\le h+b$. The aggregated estimate $\hat{\alpha }(t_0)$ for $t_0 \in [0,t^{*}]$ is computed using (4). Likewise, we also estimate $\dot{\alpha }(t_0)$ for $t_0 \in [0,t^{*}]$ using the aggregation $ \hat{\dot{\alpha }}(t_0)= n^{-1}\sum _{i=1}^{n} \hat{\dot{\alpha }}(t_0,U_i(t_0))$, where $\hat{\dot{\alpha }}(t_0,u_0)$ include the elements of $\hat{\vartheta }^{*}(t_0,u_0)$ corresponding to the position of $\dot{\alpha }(t_0)$ in $\vartheta ^{*}(t_0,u_0)$.
Step 2. We estimate $\alpha (t_{k+1})$ using the recursive formula $\hat{\alpha }(t_{k+1}) = \hat{\alpha }(t_{k})+\Delta t \hat{\dot{\alpha }}(t_{k})$, where $\hat{\alpha }(t_{k})$ and $\hat{\dot{\alpha }}(t_{k})$ are the current estimates $\alpha (t_k)$ and $\dot{\alpha }(t_k)$. Suppose that $t_{k_0}$ is the last grid point before $t^{*}$. Let $\hat{\alpha }(t_{k_0})$ and $\hat{\dot{\alpha }}(t_{k_0})$ be the estimates computed from the first step. Then $\alpha (t_{k_0+1})$ is estimated by $\hat{\alpha }(t_{k_0+1}) = \hat{\alpha }(t_{k_0})+\Delta t\hat{\dot{\alpha }}(t_{k_0})$. For $k=k_0+1, k_0+2,$ and so on, the recursive formula is used to estimate $\alpha (t_{k+1})$ with the current estimate $\hat{\alpha }(t_{k})$ and by estimating $\dot{\alpha }(t)$ at the grid points $t_k$ using the following profile procedure with the plugged-in $\hat{\alpha }(t_{k})$.
  Let $t_0=t_k$ and $u_0$ be one of the grid points in ${\cal U}^o$. Initially, we separate $\alpha (t_0)$ from $\vartheta ^{*}(t_0,u_0)$ in notations. Let $\vartheta ^{*}(t_0,u_0)=(\alpha ^{{ \mathrm{\scriptscriptstyle T} }}(t_0),\vartheta ^{**{ \mathrm{\scriptscriptstyle T} }}(t_0,u_0))^{{ \mathrm{\scriptscriptstyle T} }}$ and $\widetilde{X}_i^{*}(t|t_0,u_0)=({X_i(t)}^{{ \mathrm{\scriptscriptstyle T} }},\widetilde{X}_i^{**{ \mathrm{\scriptscriptstyle T} }}(t|t_0,u_0))^{{ \mathrm{\scriptscriptstyle T} }}$, where $\vartheta ^{**{ \mathrm{\scriptscriptstyle T} }}(t_0,u_0)=(\gamma ^{{ \mathrm{\scriptscriptstyle T} }}(u_0),\dot{\alpha }^{{ \mathrm{\scriptscriptstyle T} }}(t_0),\dot{\gamma }^{{ \mathrm{\scriptscriptstyle T} }}(u_0))^{{ \mathrm{\scriptscriptstyle T} }}$ and $\widetilde{X}_i^{**{ \mathrm{\scriptscriptstyle T} }}(t|t_0,u_0)=(Z_{i}^T(t)$, ${X_i(t)}^{{ \mathrm{\scriptscriptstyle T} }}(t-t_0),Z_{i}^T(t)$  $(U_i(t)-u_0))^{{ \mathrm{\scriptscriptstyle T} }}$. Let $\lambda _i^{**}(t, \alpha , \vartheta ^{**}|t_0,u_0)=\varphi \lbrace \alpha (t)^{{ \mathrm{\scriptscriptstyle T} }}{X_i(t)}+\vartheta ^{**{ \mathrm{\scriptscriptstyle T} }}(t_0,u_0)\widetilde{X}_i^{**}(t|t_0,u_0)\rbrace$. Then, the log-likelihood (2) can be represented as
  \begin{eqnarray*}
l(\alpha , \vartheta ^{**}|t_0,u_0)&=& \sum _{i=1}^n \int _0^\tau K_h(t-t_0)K_b(U_i(t)-u_0)\\
&&\times\, \lbrace \log (\lambda _i^{**}(t, \alpha ,\vartheta ^{**}|t_0,u_0))dN_i(t) \\
&& -\, Y_i(t) \lambda _i^{**}(t, \alpha ,\vartheta ^{**}|t_0,u_0)dt \rbrace .
\end{eqnarray*}
  Plugging $\hat{\alpha }(t_0)$ for $\alpha (t_0)$ in $l(\alpha , \vartheta ^{**}|t_0,u_0)$, this likelihood is maximized with respect to $\vartheta ^{**}$ with the estimates $\hat{\vartheta }^{**}(t_0,u_0)=(\hat{\gamma }^{{ \mathrm{\scriptscriptstyle T} }}(t_0, u_0),\hat{\dot{\alpha }}^{{ \mathrm{\scriptscriptstyle T} }}(t_0,u_0),$  $\hat{\dot{\gamma }}^{{ \mathrm{\scriptscriptstyle T} }}(t_0,u_0))^{{ \mathrm{\scriptscriptstyle T} }}$ for every grid point $u_0$. The aggregated profile estimate of $\dot{\alpha }(t_0)$ is given by $ \hat{\dot{\alpha }}(t_0)= n^{-1}\sum _{i=1}^{n} \hat{\dot{\alpha }}(t_0,U_i(t_0))$.
Step 3. Finally, $\gamma (u_0)$ is estimated by the aggregated estimator $\hat{\gamma }(u_0)$ using (4) based on $\hat{\gamma }^{{ \mathrm{\scriptscriptstyle T} }}(t_0, u_0)$ from Step 2.

In practice, although one may consider choosing the maximum possible value of $t^{*}$ such that $t^{*} \le h + b$ to incorporate more information, we find that setting $t^{*} = (h + b)/2$ provides a more stable performance in our simulations. This choice strikes a balance between information utilization and numerical stability, and it avoids potential identifiability issues that may arise when $t^{*}$ is chosen too close to the boundary.

### 2.4 Selections of kernel functions and bandwidths

We employ local linear techniques to estimate the coefficients $\alpha (t)$ and $\gamma (u)$ nonparametrically. The kernel functions are designed to give greater weight to observations near $t$ and $u$ than those further away. In the kernel smoothing literature, it has been shown that the Epanechnikov kernel function $K(x)={3}/{4}(1-x^2)I\lbrace |x| \le 1\rbrace$ has some good theoretical properties (Epanechnikov, 1969; Fan and Gijbels, 1996). We use the Epanechnikov kernel for both the kernels $K_1$ and $K_2$ in numerical studies. Silverman (1986, p.43) showed that efficiency does not vary much with the choice of kernel function: the asymptotic relative efficiency of the Tukey kernel function $K(x)={15}/{16}(1-x^2)^2I\lbrace |x| \le 1\rbrace$ compared to the optimal Epanechnikov kernel is 99%, the Gaussian kernel has a relative efficiency of 95% and the rectangular kernel has a relative efficiency about 93%.

Selecting an appropriate bandwidth, on the other hand, is crucial in the performance of nonparametric estimation as it balances the bias-variance trade-off in the estimated function (Fan and Gijbels, 1996). A bandwidth that is too small may result in overly complex models with high variance (overfitting), while a bandwidth that is too large may lead to overly smooth models with high bias (underfitting). $K$-fold cross-validation is a widely used method for estimating the prediction accuracy of a model and selecting model hyperparameters (such as the bandwidth in kernel smoothing) by splitting the data into $K$ subsets (folds) (Hastie et al., 2009). The model is trained on $K-1$ folds and tested on the remaining fold, and this process is repeated $K$ times with different combinations of training and testing sets. The average prediction accuracy across all folds is then used to evaluate the model’s performance. Let $(G_1, G_2, \cdots , G_K)$ be $K$ approximately equally-divided subsamples. We define the $k$th prediction accuracy for the test data $G_k$, $\mathrm{ACC}_k(h,b)$, based on the estimated log-likelihood function (Tian et al., 2005):


(6) \begin{eqnarray*}
\mathrm{ACC}_k(h,b)&=&\sum _{i \in G_k} \int _{t_1}^{t_2} \lbrace \log (\widehat{\lambda }_i^{(-k)}(t))dN_i(t)\\
&& -\, Y_i(t) \widehat{\lambda }_i^{(-k)}(t)dt \rbrace ,
\end{eqnarray*}

where $\widehat{\lambda }_i^{(-k)}(t)=g^{-1}\lbrace (\widehat{\alpha }^{(-k)}(t))^{ \mathrm{\scriptscriptstyle T} }X_i(t)+(\widehat{\gamma }^{(-k)}(U_i(t)))^{ \mathrm{\scriptscriptstyle T} }Z_i(t)\rbrace$, $\widehat{\alpha }^{(-k)}(\cdot )$, $\widehat{\gamma }^{(-k)}(\cdot )$ are the local linear estimators based on the training data excluding $G_k$, and $[t_1,t_2]$ is a subinterval of $(0,\tau )$. A higher estimated log-likelihood is considered having higher prediction accuracy. Then, the $K$-fold cross-validation selection of bandwidths $(h^{*}, b^{*})$ maximizes the overall prediction accuracy $\mathrm{ACC}(h,b)=\sum _{k=1}^K \mathrm{ACC}_k(h,b)$, ie, $(h^{*}, b^{*})=\arg \max _{h,b} \mathrm{ACC}(h,b).$ This procedure may be repeated a number of times, say 10 times, to reduce variability. The bandwidths are selected to maximize the average of $\mathrm{ACC}(h,b)$ over 10 repetitions.

## 3 ASYMPTOTIC PROPERTIES

In this section, we investigate the large-sample properties of the proposed estimators. Let $\alpha _0(\cdot )$ and $\gamma _0(\cdot )$ be the true vectors of functions. Let ${\cal I}_1=\lbrace {\cal I}_{jk}\rbrace$ be a $p_1\times (p_1+p_2)$ matrix with ${\cal I}_{jk}=1$ for $j=1,\dots ,p_1$ and $k=j$, and ${\cal I}_{jk}=0$ otherwise. Let ${\cal I}_2=\lbrace {\cal I}_{jk}\rbrace$ be a $p_2\times (p_1+p_2)$ matrix with ${\cal I}_{jk}=1$ for $j=1,\dots ,p_2$ and $k=j+p_1$, and ${\cal I}_{jk}=0$ otherwise. Define $\widetilde{X}_i(t)=({X}_{i}(t)^{{ \mathrm{\scriptscriptstyle T} }}, Z_{i}(t)^{{ \mathrm{\scriptscriptstyle T} }})^{{ \mathrm{\scriptscriptstyle T} }}$, $\hat{\lambda }_{i}(t)=\varphi \big \lbrace \hat{\alpha }(t)^{{ \mathrm{\scriptscriptstyle T} }}X_i(t)+ \hat{\gamma }(U_i(t))^{{ \mathrm{\scriptscriptstyle T} }}Z_i(t)\big \rbrace$, and $\hat{\dot{\lambda }}_{i}(t)=\dot{\varphi }\big \lbrace \hat{\alpha }(t)^{{ \mathrm{\scriptscriptstyle T} }}X_i(t)+ \hat{\gamma }(U_i(t))^{{ \mathrm{\scriptscriptstyle T} }}Z_i(t)\big \rbrace$. Let $\lambda _{i,0}(t,u)=\varphi \big \lbrace \alpha _0(t)^{{ \mathrm{\scriptscriptstyle T} }}X_i(t)+ \gamma _0(u)^{{ \mathrm{\scriptscriptstyle T} }}Z_i(t)\big \rbrace$, $\dot{\lambda }_{i,0}(t,u)=\dot{\varphi }\big \lbrace \alpha _0(t)^{{ \mathrm{\scriptscriptstyle T} }}X_i(t)+ \gamma _0(u)^{{ \mathrm{\scriptscriptstyle T} }}Z_i(t)\big \rbrace$, and $\ddot{\lambda }_{i,0}(t,u)=\ddot{\varphi }\big \lbrace \alpha _0(t)^{{ \mathrm{\scriptscriptstyle T} }}X_i(t)$  $+ \gamma _0(u)^{{ \mathrm{\scriptscriptstyle T} }}Z_i(t)\big \rbrace$. Then $M_i(t)=N_i(t)-\int _0^tY_i(s) \lambda _{i,0}(s,U_i(s))ds$ is a martingale with respect to the filtration $\mathcal {F}_{t}$, $0\le t\le \tau$, under independent censoring assumption. We also define $D(t,u)=E\big [Y_i(t)\big ({\dot{\lambda }_{i,0}(t,u)^2}/{{\lambda _{i,0}}(t,u)}\big )\widetilde{X}_i(t)^{\otimes 2}\big |U_i(t)=u\big ]f_{U}(t,u),$ where $f_{U}(t,u)$ is the density function of the process $U_i(t)$ at $t$ and $U_i(t)=u$.

The following theorems establish the uniform consistency and weak convergence of the proposed estimators for $t\in [t_1,t_2]\subset (0,\tau )$ and $u\in [u_1,u_2] \subset {\cal U}^o$. The proofs of the theorems are nontrivial due to the kernel smoothing in both time and time-dependent covariates. The asymptotic results related to the double kernel estimation and the martingale central limit theorems are utilized in the proofs of the theorems. The details are given in Web Appendix A of Supporting Information. Let $\Vert \cdot \Vert$ stand for Euclidean norm, $\overset{\mathcal {P}}{\rightarrow }$ for converging in probability and $\overset{\mathcal {D}}{\rightarrow }$ for converging in distribution. The conditions C.1–C.5 are given in Web Appendix A of Supporting Information.

Theorem 1: Under the conditions C.1–C.5, we have

$\displaystyle \sup _{t\in [t_1,t_2]} \Vert \hat{\alpha }(t)-\alpha _0(t) \Vert \overset{\mathcal {P}}{\rightarrow } 0$;
$\displaystyle \sqrt{nh} (\hat{\alpha }(t)-\alpha _0(t)-\frac{1}{2}h^2\mu _2\ddot{\alpha }(t)) \overset{\mathcal {D}}{\rightarrow } N\left(0, \Sigma _\alpha (t) \right)$, for $t\in [t_1,t_2]$,

where $\mu _2=\int u^2K(u)\, du$, $\nu _0=\int K^2(u)\, du$ and

\begin{eqnarray*}
\Sigma _\alpha (t)=\nu _0 E \left[Y_i(t)\frac{\dot{\lambda }_{i,0}(t,U_i(t))^2}{\lambda _{i,0}(t,U_i(t))} \lbrace {\cal I}_1 D^{-1}(t,U_i(t))\widetilde{X}_i(t) \rbrace ^{\otimes 2} \right].
\end{eqnarray*}

The covariance matrix $\Sigma _\alpha (t)$ can be consistently estimated by


\begin{eqnarray*}
\hat{\Sigma }_\alpha (t)&=&\frac{h}{n}\sum _{i=1}^n \int _0^\tau K_h(s-t)^2 \frac{\hat{\dot{\lambda }}_{i}(s)^2}{\hat{{\lambda }}_{i}(s)^2}\\
&&\times\, \lbrace {\cal I}_1\hat{D}^{-1}(t,U_i(s))\widetilde{X}_i(s) \rbrace ^{\otimes 2}dN_i(s),
\end{eqnarray*}

where


\begin{eqnarray*}
\hat{D}(t,u)&=& \frac{1}{n}\sum _{i=1}^{n}\int _0^\tau K_h(t-t)K_b(U_i(t)-u)Y_i(t)\\
&&\times\,\frac{\hat{\dot{\lambda }}_{i}(t)^2}{\hat{\lambda }_{i}(t)} \widetilde{X}_i(t)^{\otimes 2}dt.
\end{eqnarray*}

Theorem 2: Under the conditions C.1–C.5, we have

$\displaystyle \sup _{u \in [u_1,u_2]}\Vert \hat{\gamma }(u)-\gamma _0(u)\Vert \overset{\mathcal {P}}{\rightarrow } 0$;
$\displaystyle \sqrt{nb} (\hat{\gamma }(u)-\gamma _0(u)- \frac{1}{2}b^2\mu _2\ddot{\gamma }(u))\overset{\mathcal {D}}{\rightarrow } N\left(0, \Sigma _\gamma (u) \right)$, for $u \in [u_1,u_2]$,

where

\begin{eqnarray*}
\Sigma _\gamma (u)&=&\lim _{n\rightarrow \infty } bE\Big [\int _0^\tau K_b(U_i(t)-u)^2Y_i(t)\frac{\dot{\lambda }_{i,0}(t,U_i(t))^2}{\lambda _{i,0}(t,U_i(t))}\\
&&\times\, \lbrace {\cal I}_2 D^{-1}(t,u)\widetilde{X}_i(t) \rbrace ^{\otimes 2}dt\Big ].
\end{eqnarray*}

The asymptotic covariance matrix $\Sigma _\gamma (u)$ can be consistently estimated by


\begin{eqnarray*}
\hat{\Sigma }_\gamma (u)&=& \frac{b}{n}\sum _{i=1}^n \int _0^\tau K_b(U_i(t)-u)^2 \frac{\hat{\dot{\lambda }}_{i}(t)^2}{\hat{{\lambda }}_{i}(t)^2} \\
&&\times\,\lbrace {\cal I}_2 \hat{D}^{-1}(t,u)\widetilde{X}_i(t) \rbrace ^{\otimes 2}dN_i(t).
\end{eqnarray*}

## 4 SIMULATION STUDIES

### 4.1 Model generation and settings

We conducted an extensive simulation study to evaluate the finite sample properties of the proposed estimators. Simulation of recurrent event processes under model (1) is nontrivial. We used the thinning method of Lewis and Shedler (1979), which can be used to simulate inhomogeneous Poisson processes by “thinning” the points from the homogeneous versions. Algorithm 1 in the following outlines the data-generation steps for simulating recurrent events with intensity function given by model (1) for $U_{i}(t)=t-T_{i,{N_{i}(t^-)}}$ and $Z_i(t)=W_i(t)I(N_i(t^-)>0)$. The algorithm for simulating recurrent event processes from models with different $U_{i}(t)$ and $Z_i(t)$ can be readily modified based on this algorithm.


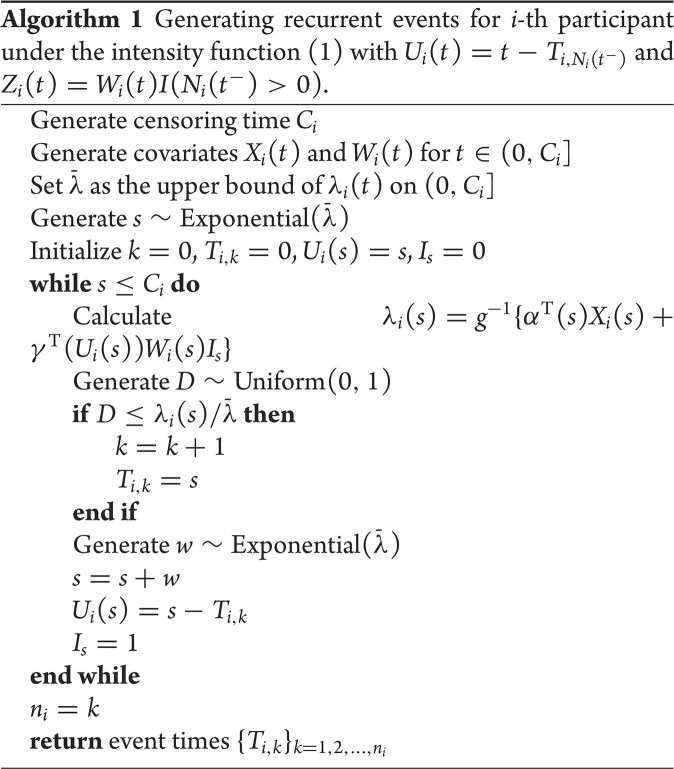


We present the simulation results for two model settings under model (1). In each of the settings, we consider both additive intensity and multiplicative intensity models. We also take $U_{i}(t)=t-T_{i,{N_{i}(t^-)}}$, which is the time elapsed from the previous event, for both settings. The model for Simulation I presented in Section 4.2 has two covariates $X_i$ and $W_i$. Thus, $\alpha (t)$ and $\gamma (u)$ are the temporal covariate effects in two different time scales. The estimation procedure based on the local score estimating equation (3) and the aggregation formula (4) can be directly used for the nonparametric estimation. The model for Simulation II in Section 4.3 does not involve any covariates, which is a special scenario of overlapping covariates $X_i(t) = W_i(t) = 1$. The adaptive algorithm needs to be adopted to estimate parameters nonparametrically to avoid identifiability issue in certain local regions. The Epanechnikov kernel $K(x)={3}/{4}(1-x^2)I\lbrace |x| \le 1\rbrace$ is used for numerical studies. We use fixed bandwidths $(h,b)=(0.3,0.3)$ in the simulations for all scenarios for computational feasibility. The proposed $K$-fold cross-validation bandwidth procedure is used in the data application in Section 5.

To evaluate the performance of the proposed estimators for $\alpha (t)$ and $\gamma (u)$, we conducted simulations for three different sample sizes based on 500 repetitions and reported biases (Bias), empirical standard errors (SEE), average estimated standard errors (ESE), and the 95% pointwise coverage probabilities (CP) for each simulation model.

### 4.2 Simulation I

In this subsection, we examine the performance of the proposed estimation methods when $X_i$ and $W_i$ do not have overlapping components. We let $X_i$ follow a Bernoulli distribution with probability of success $p=0.5$ and $W_i$ a uniform random variable on [0,1]. We considered two link functions, the logarithm link and the identity link, which yield the following multiplicative intensity model and additive intensity model, respectively,


(7) \begin{eqnarray*}
\lambda _i(t)&=&\exp \lbrace {\alpha _0}(t)+{\alpha _1}(t)X_i\\
&&+\,{\gamma }(t-T_{N_i(t^-)})I(N_i(t^-)> 0)W_i \rbrace ,
\end{eqnarray*}

(8) \begin{eqnarray*}
\lambda _i(t)={\alpha _0}(t)+{\alpha _1}(t)X_i+{\gamma }(t-T_{N_i(t^-)})I(N_i(t^-){>}0)W_i,\\
\end{eqnarray*}

for $0\le t\le \tau$. The censoring time $C_i$ is set as the minimum of $\tau =4$ and $C_i^{*}$, where $C_i^{*}$ is generated from the Uniform(3,8) distribution.

**FIGURE 2:**
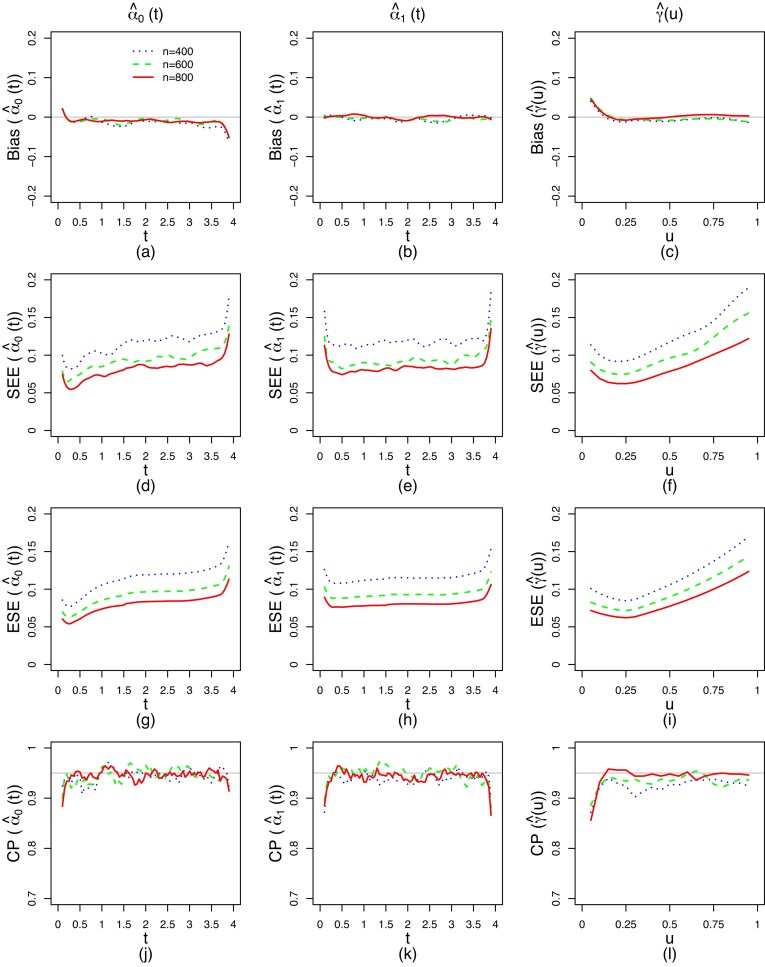
Estimation results for $\hat{\alpha }_0(t)$, $\hat{\alpha }_1(t)$ and $\hat{\gamma }(u)$ under model (7). In each panel (left for $\hat{\alpha }_0(t)$, middle for $\hat{\alpha }_1(t)$, and right for $\hat{\gamma }(u)$), lines represent different sample sizes: blue dotted for $n=400$, green dashed for $n=600$, and red solid for $n=800$. The results are based on 500 repetitions. Bias, SEE, ESE, and CP stand, respectively, for the bias, empirical standard error, average estimated standard errors, and 95% empirical coverage probabilities.

For the multiplicative intensity model (7), we set $\alpha _0(t)=1-\log (1+0.2\log (1+t))$, $\alpha _1(t)=-0.5+0.1t$, and $\gamma (u)=-0.3/(1+u)$. The average number of recurrent events per participant is around 4 for $X_i=1$ and roughly 9 for $X_i=0$. Figure 2 summarizes Bias, SEE, ESE and 95% CP of the proposed estimators $\hat{\alpha }_0(t)$, $\hat{\alpha }_1(t)$, and $\hat{\gamma }(u)$ under model (7) for different sample sizes $n=400, 600$ and 800. The estimators exhibit small bias, with estimated standard errors closely aligned with empirical standard errors. Furthermore, the 95% empirical CP consistently hover around the nominal level, underlining the adequacy of the estimators for the model parameters and their variance estimators.

For the additive intensity model (8), we used $\alpha _0(t)=4-\log (1+t)$, $\alpha _1(t)=-1+0.2t$, and $\gamma (u)=-1/(1+u)$. Notably, the average number of recurrent events for each participant is about 6 for $X_i=1$ and approximately 9 for $X_i=0$. Web Figure 1 in the Supporting Information shows that the proposed estimators perform well under the additive intensity model.

### 4.3 Simulation II

**FIGURE 3:**
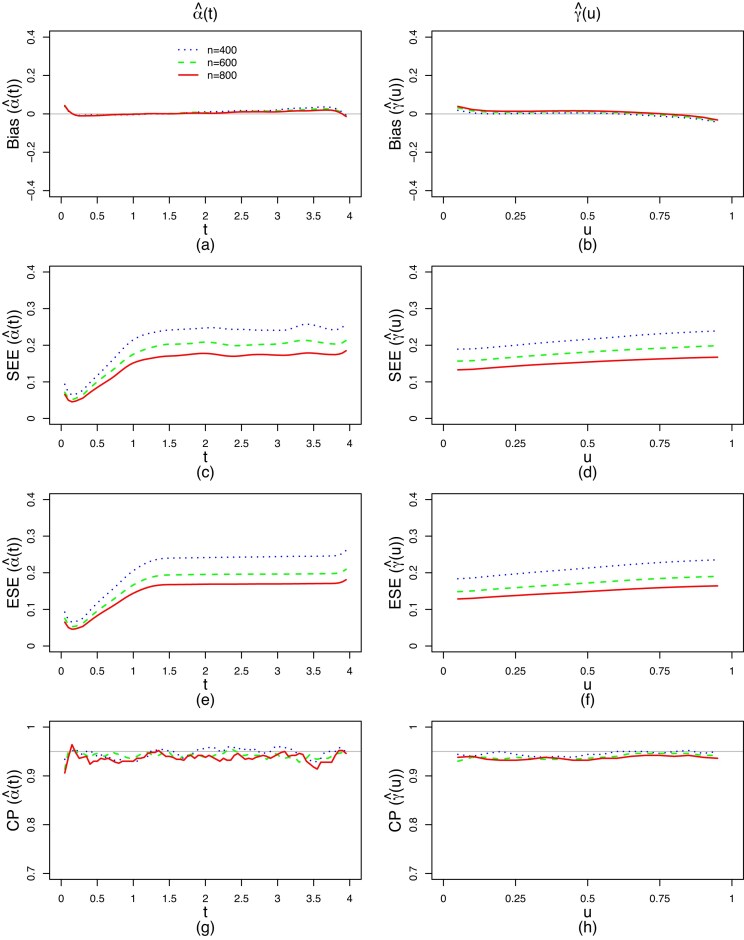
Estimation results for $\hat{\alpha }(t)$ and $\hat{\gamma }(u)$ under model (9). In each panel (left for $\hat{\alpha }(t)$ and right for $\hat{\gamma }(u)$), lines represent different sample sizes: blue dotted for $n=400$, green dashed for $n=600$, and red solid for $n=800$. The results are based on 500 repetitions. Bias, SEE, ESE, and CP stand, respectively, for the bias, empirical standard error, average estimated standard errors, and 95% empirical coverage probabilities.

In this subsection, we examine the performance of the proposed adaptive algorithm when $X_i$ and $W_i$ have overlapping components. The simulation sets $X_i(t) = W_i(t) = 1$. First, we conducted a simulation study for the multiplicative intensity model:


(9) \begin{eqnarray*}
\lambda _i(t)=\exp \big \lbrace {\alpha }(t)+{\gamma }(t-T_{N_i(t^-)})I(N_i(t^-)> 0)\big \rbrace ,
\end{eqnarray*}

where $\alpha (t)=1-\log (1+0.2\log (1+t))$ and $\gamma (u)=-2(1-u)/\exp ((1-u)^2)$. On average, each participant experienced approximately 5 events. As discussed in Section 2.2, the identifiability of coefficient functions ${\alpha }(t)$ and ${\gamma }(u)$ can be challenging in certain local regions when covariates $X_i(t)$ and $W_i(t)$ share common vectors. To address this, we employed the adaptive algorithm to estimate these parameters. The standard errors were estimated using the bootstrap method with 500 bootstrap samples. The bootstrap sampling was conducted at the participant level, not the event level. In other words, for each bootstrap iteration, we randomly sampled participants (with replacement) rather than recurrent events. The results of the simulation, including the estimators obtained through the adaptive algorithm and bootstrap-estimated standard errors, demonstrate satisfactory finite-sample performance. These results are depicted in Figure 3.

In addition, we conducted a simulation study for the additive intensity model:


(10) \begin{eqnarray*}
\lambda _i(t)={\alpha }(t)+{\gamma }(t-T_{N_i(t^-)})I(N_i(t^-)> 0),
\end{eqnarray*}

where $\alpha (t)=4-\log (1+t)+0.3t$ and $\gamma (u)=-0.5/(1+u)$. The simulation results shown in Web Figure 2 in the Supporting Information similarly showcased satisfactory performance, affirming the effectiveness of the adaptive algorithm in handling overlapping covariates across different model specifications.

We conducted an additional simulation study that expands the covariate structure in both $X_i(t)$ and $W_i(t)$ beyond the intercept-only case. The adaptive estimation procedure continues to perform well in this more complex scenario. A summary of the simulation results is provided in Web Appendix C.

## 5 APPLICATION TO THE MALARIA VACCINE TRIAL

The malaria vaccine trial MAL-094 conducted at the Agogo, Ghana and Siaya, Kenya study sites enrolled children aged 5-17 months without serious acute or chronic illness who had previously received three doses of diphtheria, tetanus, pertussis, and hepatitis B vaccine and at least three doses of oral polio vaccine (Samuels et al., 2022; Westercamp et al., 2024). 1500 children were evenly randomly allocated to a rabies control vaccine or to one of four RTS,S/AS01$_{\rm E}$ vaccine arms with different vaccination and dosage schedules (Juraska et al., 2024). Blood samples for efficacy analyses were taken at scheduled monthly visits up to month 20 and at 3-monthly intervals between month 20 and month 32 at study clinics or participants’ households.

Most malaria vaccine trials evaluate vaccine efficacy using clinical disease as an outcome. However, a large proportion of malaria infections are asymptomatic. Asymptomatic individuals remain infectious to mosquitoes, and thus act as silent reservoirs of transmission (Galatas et al., 2016). This is one of the challenges posed to control and elimination of malaria disease. The MAL-095 sub-study of MAL-094 was a genotyping study of dried blood spot samples designed to enhance understanding of vaccine protection by analyzing molecularly detected new infections Juraska et al. (2024). By applying deep sequencing of malaria parasites to all dried blood spot samples collected 4-weekly for 20 months and 3-monthly for 12 months from 1500 participants, the MAL-095 sub-study ascertained recurrent new malaria infections defined by new genetic variants. In particular, Illumina-based amplicon sequencing of the circumsporozoite protein C-terminus coding region and a comparably polymorphic coding region for the antigen serine repeat antigen 2 was applied to DNA extracted from each dried blood spot sample. From these sequence data at a given sampling time point, distinct haplotypes were defined as the combined genotype of all nucleotide variants in each amplicon sequence. Then, a new malaria infection at a specific sampling date is defined by at least one haplotype observed for either amplicon that had not been previously detected in the preceding three sample timepoints from that individual. This molecular detection of new malaria infection does not depend on whether the infection was symptomatic.

**FIGURE 4:**
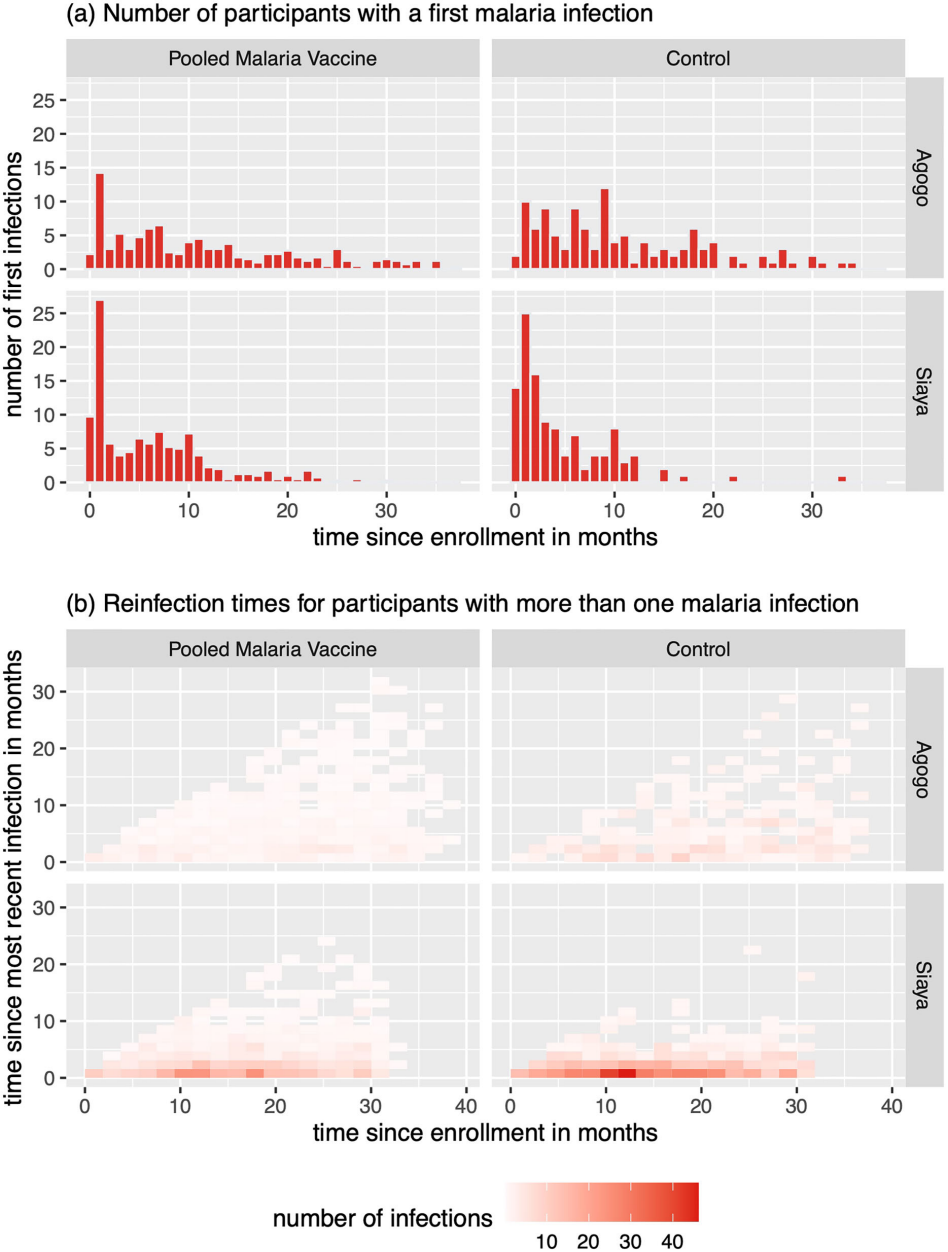
Temporal dynamics of infections and re-infections by treatment arm and study site. The horizontal axis denotes time since enrollment, while the vertical axis denotes time since the most recent infection. Color in figure (b) reflects the number of infections within a specific time block defined by the two time axes.

We apply the method developed in Section 2 to dynamically model the intensity of the molecularly detected new malaria infections, which addresses Exploratory objective 2 of the MAL-05 Statistical Analysis Plan. A participant is considered censored if the participant missed three consecutive scheduled visits and with no intervening unscheduled visits in-between in which case the censoring time is defined as the time of the last follow-up visit which is taken to be Month 32. By Month 32, 4633 new malaria infections were observed before censoring among 1461 participants, with 1065 having experienced at least one infection. For those with a history of infection, the average number of new infections experienced during the follow-up period was 4.4. The heat map (Figure 4) illustrates the temporal evolution of re-infections (second or successive infections) in both the pooled vaccine arm and the control arm by Agogo and Siaya in the two time scales: time since enrollment and time since the last infection. The heat map indicates that the risk of re-infection for participants varies over time, the pooled malaria vaccine exhibits a lower risk of re-infection compared to the control arm, and the Siaya site has higher risk than the Agogo site.

To assess effectiveness of the RTS,S/AS01$_{\rm E}$ vaccine to protect against new infections, other risk factors of re-infections and how the risk of re-infections are affected by previous infections, we consider the following multiplicative temporal intensity model. For participant $i$, the conditional intensity is postulated as


(11) \begin{eqnarray*}
\lambda _i(t)&=&\exp \big \lbrace \alpha _0(t){+}\alpha _1(t)\mathrm{Vacc}_i{+}\alpha _2(t)\mathrm{Agogo}_i+\alpha _3(t)\mathrm{Age}_i \\
&&+\,\gamma _0(t-T_{N_i(t^-)})I(N_i(t^-)> 0)\\
&&+\,\gamma _1(t-T_{N_i(t^-)})I(N_i(t^-)>0)\mathrm{Vacc}_i\big \rbrace
\end{eqnarray*}

for $t\in [0,32]$ (months), where $\mathrm{Vacc}_i$ is the pooled malaria vaccine indicator ($\mathrm{Vacc}_i=1$ if assigned to one of the four RTS,S/AS01$_{\rm E}$ vaccine arms, 0 if assigned to the control arm), $\mathrm{Agogo}_i$ is the study site indicator (1= Agogo, 0 = Siaya), and $\mathrm{Age}_i$ is the age in months at enrollment. The bandwidths $h=11, b=5.667$ (months) are selected via 5-fold cross-validation with 10 repetitions as described in Section 2.4. The plot of the total prediction accuracy against $(h,b)$ is shown in Web Figure 4.

**FIGURE 5:**
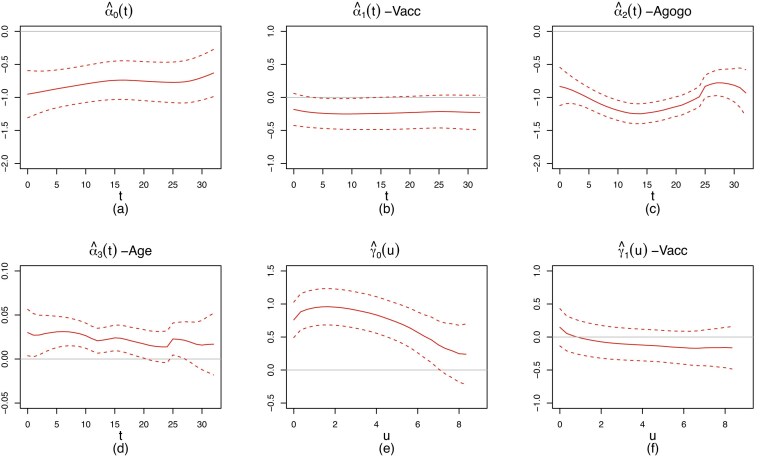
Estimation of temporal effects of covariates on the malaria infection intensity under the multiplicative temporal intensity model (11), where $t$ is the time since enrollment and $u$ is the time elapsed since most recent infection. The solid line represents the point estimate, while the dashed lines signify the 95% pointwise confidence band.

Figure 5 presents the estimated (calendar) time-varying effects $\lbrace \hat{\alpha }_k(t), k=0,1,2,3\rbrace$ and the estimated (backward recurrence) time-varying effects $\lbrace \hat{\gamma }_k(u), k=0,1\rbrace$. The $\hat{\gamma }_k(u)$’s are plotted on $u\in [0,8.33]$, where 8.33 months is the 90th percentile of the gap times observed for re-infections. As we delve into these temporal effects, we observe an increase in the baseline infection intensity over calendar time. As the study progressed, obviously the children increased in age. The baseline infection intensity trend aligns with the knowledge that older children have a higher risk of infections. The infection risk in the pooled malaria vaccine arm is lower than that in the control arm. Participants living at the Agogo site have significantly lower risk of new infection than those living at Siaya. This aligns with the finding that before the start of the MAL094 study, Kenya had a prevalence approximately double that of Ghana (39% versus 17%, as estimated by microscopy) (Samuels et al., 2022). Following prior infections, the risk of a new infection increases in the control arm for a child who already had the first new infection compared to a child who had not had a new infection (shown with $\hat{\gamma }_0(u)>0$), but this increment in risk appears to decline over time (shown with $\hat{\gamma }_0(u)$ decreasing in $u$). The risk of subsequent new infections also appears higher in the pooled malaria vaccine arm (shown with $\hat{\gamma }_0(u)+\hat{\gamma }_1(u)>0$). But the increased risk of re-infections is lower in the pooled malaria vaccine arm than in the control arm (with $\hat{\gamma }_1(u)< 0$).

To quantify the level of protection against re-infection, we define the vaccine efficacy (VE) as the percentage reduction in infection intensity of malaria vaccinated individuals compared to those who were rabies vaccinated. Under model (11), the VE at time $t$ equals


\begin{eqnarray*}
\mathrm{VE}(t) &=& 1-\frac{{\lambda }_i(t|\mathrm{Vacc}_i=1)}{{\lambda }_i(t|
\mathrm{Vacc}_i=0)}\\
&=& 1-\exp \lbrace \alpha _1(t)+\gamma _1(t-T_{N_i(t^-)}) I(N_i(t^-)>0) \rbrace .
\end{eqnarray*}

Note that $\mathrm{VE}(t) =1-\exp \lbrace \alpha _1(t) \rbrace$ is vaccine efficacy against the first infection and $\mathrm{VE}(t) =1-\exp \lbrace \alpha _1(t)+\gamma _1(t-T_{N_i(t^-)}) \rbrace$ is vaccine efficacy against re-infections.

**FIGURE 6:**
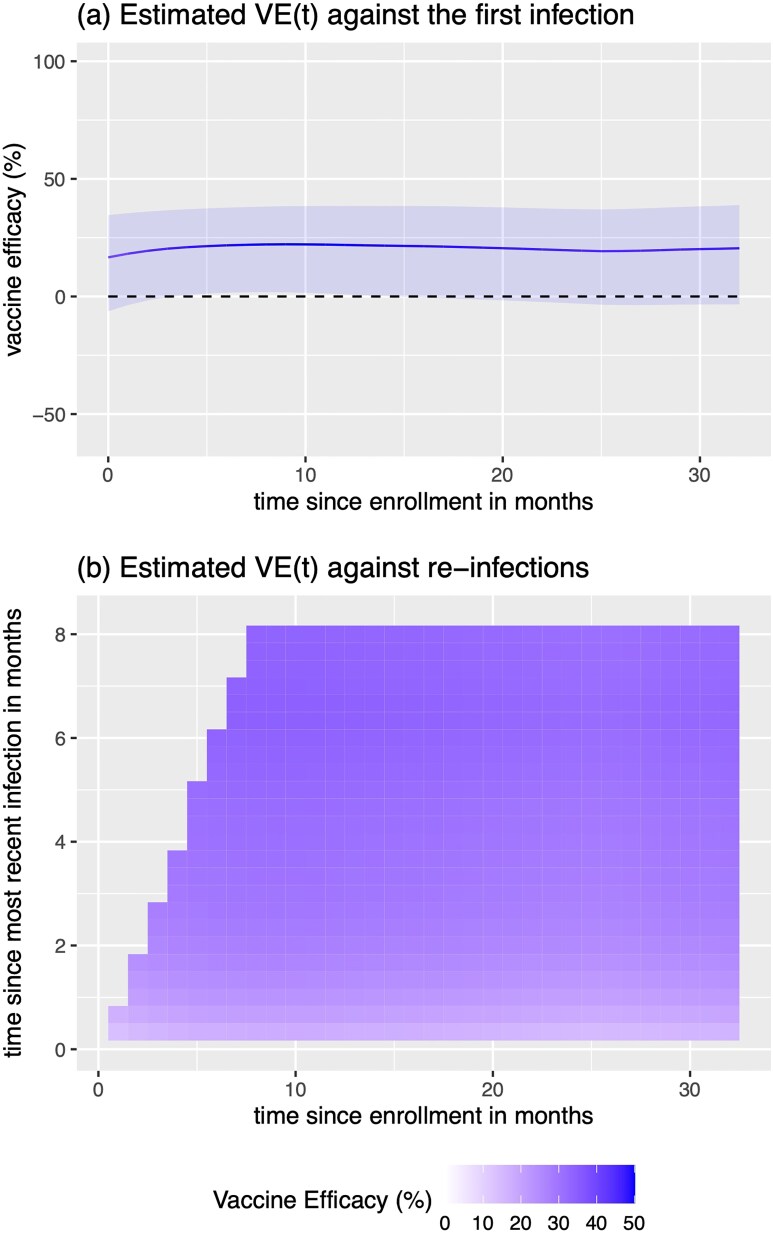
The estimated vaccine efficacy against the first infection (a) and against the re-infection (b) under the multiplicative temporal intensity model (11).

Figure 6 shows the estimated VE against the first infection in (a) and against re-infection in (b). The estimated VE against the first infection shown in Figure 6 (a) is about 20% with 95% confidence band ranging from 0 to about 38%. Figure 6 (b) shows a heat map of the estimated VE in two time-scales: time since enrollment and time since last new malaria infection for participants who became infected. We observe that VE increased with the time since last infection. VE against re-infections is low in a short-term post-infection but it increased to the range between 30%–40% in 4 to 8 months after the most recent infection. A possible interpretation is that the participants in both the malaria vaccine and control arms generate anti-malaria antibodies after an infection. The difference in antibody levels between the malaria vaccine and the control groups over the short-term post-infection is small such that VE against re-infections is low over the short term post-infection. However, the participants in the control arm may have experienced a decline in antibody titers over time, while the participants in the malaria vaccine arm received booster shots to maintain relatively high antibody, leading to the observed temporal pattern of vaccine efficacy.

The proposed methods are applied for additional data analyses for the MAL094/MAL095 trial in Web Appendix E, where we investigate how the intensity of new malaria infection is affected by the time since the most recent vaccination.

## 6 CONCLUDING REMARKS

Motivated by the MAL-095 sub-study of the MAL-094 malaria vaccine trial, we proposed a generalized nonparametric temporal intensity model for recurrent events that integrates features of inhomogeneous Poisson and renewal-type models. This framework enables the study of how event intensity evolves over time and how prior events influence future risk, accommodating models such as multiplicative and additive intensity forms. Unlike standard Cox-type models that assume constant covariate effects, our approach captures dynamic changes in risk—essential for understanding waning vaccine efficacy. Applying our method to MAL-094/MAL-095 data provided new insights into the protective effects of the RTS,S/AS01$_{\rm E}$ vaccine and how prior infections modify future malaria risk.

While our nonparametric approach offers flexibility, it demands larger sample sizes and poses theoretical and computational challenges. Simpler parametric models, though more tractable, risk misspecification. Future work will explore semiparametric extensions to balance flexibility and interpretability.

While our methods are for right-censored data, with recurrent endpoints detection of malaria infection, if instead the investigated endpoint is malaria infection, then the endpoint is interval censored. The highly frequent and sensitive malaria diagnostic testing in MAL-095 limits the potential insights gained from such methods. Nevertheless, extending our methods to handle interval-censored data remains a significant direction for future research.

## Supplementary Material

ujaf146_Supplemental_Files Web Appendices for the proofs of the theorems, additional simulations and data analysis, the bandwidth selection procedure used for the data example, along with the computer code, referenced in Sections 3, 4, and 5 are available with this paper at the Biometrics website on Oxford Academic.

## Data Availability

The authors obtained the data from a third party and are not permitted to share the data publicly due to data use agreement.
