# Supplementary material for: Generalized nonparametric temporal modeling of recurrent events with application to a malaria vaccine trial
Source: Biometrics. 2025 Nov 21;81(4):ujaf146. doi: 10.1093/biomtc/ujaf146 (PMC12635532; doi:10.1093/biomtc/ujaf146)
Supplement: ujaf146_Supplemental_Files — Web Appendices for the proofs of the theorems, additional simulations and data analysis, the bandwidth selection procedure used for the data example, along with the computer code, referenced in Sections 3, 4, and 5 are available with this paper at the Biometrics website on Oxford Academic. [file ujaf146_supplemental_files.zip › Biometrics_Dynamic_intensity_Supp_Rev2.pdf]

Supplementary Materials for “Generalized Nonparametric Temporal Modeling of Recurrent Events with Application to a Malaria Vaccine Trial”

Fei Heng[1], Yanqing Sun[2,*], Jing Xu[3], and Peter B. Gilbert[3,4]

[1] Department of Mathematics and Statistics, University of North Florida, Jacksonville, Florida, U.S.A.,

[2] Department of Mathematics and Statistics, University of North Carolina at Charlotte, Charlotte, North Carolina, U.S.A.

[3] Vaccine and Infectious Disease, Fred Hutchinson Cancer Center, Seattle, Washington, U.S.A.

[4] Department of Biostatistics, University of Washington, Seattle, Washington, U.S.A.

This online Supplementary Materials includes Web Appendices A, B, C, D and E. Web Appendix A provides the conditions for Theorems 1 and 2 and their detailed proofs. Web Appendix B includes some technical notes for deriving the local log-likelihood function. Web Appendix C includes additional simulation results. Web Appendix D gives details of bandwidth selection for the data example. Web Appendix E presents additional data analyses for the MAL094/MAL095

*Corresponding author: yasun@charlotte.edu

trial, where we investigate how the intensity of new malaria infection is affected by the time since the most recent vaccination.

The MATLAB code for the simulation studies, along with README files providing instructions for running the code, is also a part of Supplementary Materials. The zip file contains two folds, one for Simulation I and the other for Simulation II of Section 4.

The equation numbers not given in the supplementary file are referred to in the main paper.

Web Appendix A: Conditions and proofs of theorems

The following conditions are assumed for Theorem 1 and Theorem 2.

C.1 The inverse function of the link function $\varphi(\cdot) = g^{-1}(\cdot)$ is twice differentiable.

C.2 The processes $X_i(t)$, $Z_i(t)$, $U_i(t)$ and $\lambda_i(t)$, $0 \le t \le \tau$, are left-continuous, bounded and their total variations are bounded by a constant.

C.3 The kernel function $K(\cdot)$ is symmetric with compact support on $[-1, 1]$ and Lipschitz continuous. Bandwidths $h \asymp b$; $h \to 0$; $nh^2 \to \infty$ and nh^5 is bounded.

C.4 $\alpha_0(t)$ is twice differentiable on $t \in [0, \tau]$, $\gamma_0(u)$ is twice differentiable on a compact support $\mathcal{U}$. $D^{-1}(t, u)$ is positive definite for all $(t, u) \in [0, \tau] \times \mathcal{U}$.

C.5 The density $f_U(t, u)$ is twice continuously differentiable with respect to u and satisfies $\inf_{t\in[0,\tau],u\in\mathcal{U}} f_U(t, u) > 0$.

The following two lemmas are used in the proofs of Theorem 1 and Theorem 2. Let $\vartheta_0^*(t, u) = (\alpha_0^{\mathrm{T}}(t), \gamma_0^{\mathrm{T}}(u), \dot{\alpha}_0^{\mathrm{T}}(t), \dot{\gamma}_0^{\mathrm{T}}(u))^{\mathrm{T}}$ and $\vartheta_0(t, u) = (\alpha_0^{\mathrm{T}}(t), \gamma_0^{\mathrm{T}}(u))^{\mathrm{T}}$, where $\alpha_0(\cdot), \gamma_0(\cdot), \dot{\alpha}_0(\cdot), \dot{\gamma}_0(\cdot)$ are true values of $\alpha(\cdot), \gamma(\cdot)$ and their first derivatives.

Lemma 1. *Under the conditions C.1- C.5, we have that*

$$H\hat{\vartheta}^*(t, u) \xrightarrow{\mathcal{P}} (\vartheta_0(t, u)^{\mathrm{T}}, \mathbf{0}_p)^{\mathrm{T}}$$

uniformly in $t \in [t_1, t_2], u \in [u_1, u_2]$ *as* $n \to \infty$, *where* $H = diag[I_p, hI_{p_1}, bI_{p_2}]$, $p = p_1 + p_2$, *and* $\mathbf{0}_p$ *is a* $p \times 1$ *vector of zeros.*

Proof of Lemma 1. Let $\eta(t,u) = H(\vartheta^*(t,u) - \vartheta_0^*(t,u))$. To prove $H\vartheta_0^*(t,u) \xrightarrow{\mathcal{P}} (\vartheta_0(t,u)^{\mathrm{T}}, \mathbf{0}_p)^{\mathrm{T}}$ is equivalent to showing $\hat{\eta}(t,u) = H(\hat{\vartheta}^*(t,u) - \vartheta_0^*(t,u)) \xrightarrow{\mathcal{P}} \mathbf{0}_{2p}$. In the following, for simplicity, we denote $\eta(t_0,u_0)$, $\hat{\eta}(t_0,u_0)$ and $\vartheta_0^*(t_0,u_0)$ as η, $\hat{\eta}$ and ϑ_0^* respectively. Let $\lambda_i^*(t,\theta) = \varphi\left\{\theta^{\mathrm{T}}\widetilde{X}_i^*(t|t_0,u_0)\right\}$, $\dot{\lambda}_i^*(t,\theta) = \dot{\varphi}\left\{\theta^{\mathrm{T}}\widetilde{X}_i^*(t|t_0,u_0)\right\}$, and $\ddot{\lambda}_i^*(t,\theta) = \ddot{\varphi}\left\{\theta^{\mathrm{T}}\widetilde{X}_i^*(t|t_0,u_0)\right\}$ where θ is an arbitrary $2p$-dimentional column vector. Then, by equation (3), $\hat{\eta}$ is the solution of $U_\eta(\eta|t_0,u_0) = 0$, where

$$U_\eta(\eta|t_0,u_0) = \sum_{i=1}^{n}\int_0^\tau K_h(t-t_0)K_b(U_i(t)-u_0)Y_i(t)\frac{\dot{\lambda}_i^*(t,H^{-1}\eta+\vartheta_0^*)}{\lambda_i^*(t,H^{-1}\eta+\vartheta_0^*)} \left\{dN_i(t) - \lambda_i^*(t,H^{-1}\eta+\vartheta_0^*)dt\right\}\widetilde{X}_i^*(t|t_0,u_0).$$

First, we consider

$$\begin{aligned}
&n^{-1}H^{-1}(U_\eta(\eta|t_0,u_0) - U_\eta(\mathbf{0}_{2p}|t_0,u_0))\\
&= n^{-1}\sum_{i=1}^{n}\int_0^\tau K_h(t-t_0)K_b(U_i(t)-u_0)Y_i(t)\frac{\dot{\lambda}_i^*(t,H^{-1}\eta+\vartheta_0^*)}{\lambda_i^*(t,H^{-1}\eta+\vartheta_0^*)}\\
&\left\{dN_i(t) - \lambda_i^*(t,H^{-1}\eta+\vartheta_0^*)dt\right\}H^{-1}\widetilde{X}_i^*(t|t_0,u_0)\\
&- n^{-1}\sum_{i=1}^{n}\int_0^\tau K_h(t-t_0)K_b(U_i(t)-u_0)Y_i(t)\frac{\dot{\lambda}_i^*(t,\vartheta_0^*)}{\lambda_i^*(t,\vartheta_0^*)}\\
&\left\{dN_i(t) - \lambda_i^*(t,\vartheta_0^*)dt\right\}H^{-1}\widetilde{X}_i^*(t|t_0,u_0).
\end{aligned}$$

Let $D_{22}(t_0,u_0) = D(t_0,u_0)\circ\mathrm{diag}(\mu_2\mathbb{1}_{p_1\times p_1}, \mu_2\mathbb{1}_{p_2\times p_2})$, where $\circ$ is the Hadamard product, $\mathbb{1}_{m\times n}$ is a $m\times n$ all ones matrix:

$$\begin{bmatrix}
1 & 1 & 1 & \dots & 1\\
1 & 1 & 1 & \dots & 1\\
\vdots & \vdots & \vdots & \ddots & \vdots\\
1 & 1 & 1 & \dots & 1
\end{bmatrix}_{m\times n}.$$

Under Conditions C.1-C.5, by the Taylor expansion and the Lemma A.1 in Yin et al. (2008), we have

$$\begin{aligned}
&n^{-1}H^{-1}(U_\eta(\eta|t_0,u_0)-U_\eta(\mathbf{0}_{2p}|t_0,u_0))\\
&=n^{-1}\sum_{i=1}^{n}\int_0^\tau K_h(t-t_0)K_b(U_i(t)-u_0)Y_i(t)\Big[\Big\{\frac{\ddot{\lambda}_i^*(t,\vartheta_0^*)}{\lambda_i^*(t,\vartheta_0^*)}-\frac{\dot{\lambda}_i^*(t,\vartheta_0^*)^2}{\lambda_i^*(t,\vartheta_0^*)^2}\Big\}dN_i(t)\\
&\quad-\ddot{\lambda}_i^*(t,\vartheta_0^*)dt\Big]\{H^{-1}\widetilde{X}_i^*(t|t_0,u_0)\}^{\otimes 2}\eta+o_p(\eta)\\
&=-\mathrm{diag}(D(t_0,u_0),D_{22}(t_0,u_0))\eta+o_p(\eta)
\end{aligned}$$

uniformly in $t_0\in[t_1,t_2]$, $u_0\in[u_1,u_2]$ and $\eta\in\mathcal{N}_0$, a neighborhood of $\mathbf{0}_{2p}$. Furthermore,

$$\begin{aligned}
&n^{-1}H^{-1}U_\eta(\mathbf{0}_{2p}|t_0,u_0)\\
&=n^{-1}\sum_{i=1}^{n}\int_0^\tau K_h(t-t_0)K_b(U_i(t)-u_0)Y_i(t)\frac{\dot{\lambda}_{i,0}(t,U_i(t))}{\lambda_{i,0}(t,U_i(t))}H^{-1}\widetilde{X}_i^*(t|t_0,u_0)dM_i(t)+o_p(1).
\end{aligned}$$

By applying Lemma 1 of Zhang et al. (2013), $n^{-1}H^{-1}U_\eta(\mathbf{0}_{2p}|t_0,u_0)\xrightarrow{\mathcal{P}}\mathbf{0}_{2p}$. By Lemma 2 of Sun et al. (2012), we conclude that $\hat{\eta}(t,u)\xrightarrow{\mathcal{P}}\mathbf{0}_{2p}$, thus $H\hat{\vartheta}^*(t,u)\xrightarrow{\mathcal{P}}(\vartheta_0(t,u)^{\mathrm{T}},\mathbf{0}_p)^{\mathrm{T}}$ uniformly in $t\in[t_1,t_2], u\in[u_1,u_2]$ as $n\to\infty$. □

Lemma 2. *Under the conditions C.1- C.5, we have*

$$\begin{aligned}
&\sqrt{nhb}\Big\{\hat{\vartheta}(t_0,u_0)-\vartheta_0(t_0,u_0)-\frac{1}{2}h^2\mu_2D^{-1}(t_0,u_0)b_\alpha(t_0,u_0)-\frac{1}{2}b^2\mu_2D^{-1}(t_0,u_0)b_\gamma(t_0,u_0)\Big\}\\
&=D^{-1}(t_0,u_0)\sqrt{nhb}\mathbf{A}_n(t_0,u_0)+o_p(1),
\end{aligned}$$

uniformly in $t_0\in[t_1,t_2]$ *and* $u_0\in[u_1,u_2]$ *as* $nh^6=O_p(1)$, *where*

$$\begin{aligned}
b_\alpha(t_0,u_0)=&E\Big\{Y_i(t_0)\frac{\dot{\lambda}_{i,0}(t_0,u_0)^2}{\lambda_{i,0}(t_0,u_0)}\widetilde{X}_i(t_0)X_i^{\mathrm{T}}(t_0)\Big|U_i(t_0)=u_0\Big\}f_U(t_0,u_0)\ddot{\alpha}(t_0),\\
b_\gamma(t_0,u_0)=&E\Big\{Y_i(t_0)\frac{\dot{\lambda}_{i,0}(t_0,u_0)^2}{\lambda_{i,0}(t_0,u_0)}\widetilde{X}_i(t_0)W_i^{\mathrm{T}}(t_0)I(N_i(t^-)>0)\Big|U_i(t_0)=u_0\Big\}f_U(t_0,u_0)\ddot{\gamma}(u_0),\\
\mathbf{A}_n(t_0,u_0)=&n^{-1}\sum_{i=1}^{n}\int_0^\tau K_h(t-t_0)K_b(U_i(t)-u_0)Y_i(t)\frac{\dot{\lambda}_{i,0}(t,U_i(t))}{\lambda_{i,0}(t,U_i(t))}\widetilde{X}_i(t)dM_i(t).
\end{aligned}$$

Proof of Lemma 2. Because $U(\hat{\vartheta}^*|t_0, u_0) = 0$, we have

$$\hat{\vartheta}^*(t_0, u_0) - \vartheta_0(t_0, u_0) = -\Big\{\frac{\partial U(\vartheta_0^*|t_0, u_0)}{\partial \vartheta^*}\Big\}^{-1} U(\vartheta_0^*|t_0, u_0) + o_p(1).$$

We consider the first p components of ϑ^*. Since

$$\frac{\partial U(\vartheta_0^*|t_0, u_0)}{\partial \vartheta^*} \xrightarrow{\mathcal{P}} -\mathrm{diag}(D(t_0, u_0), D_{22}(t_0, u_0)),$$

we have

$$\hat{\vartheta}(t_0, u_0) - \vartheta_0(t_0, u_0) = D^{-1}(t_0, u_0) U_1(\vartheta_0^*|t_0, u_0) + o_p(1),$$

where

$$U_1(\vartheta_0^*|t_0, u_0) = n^{-1} \sum_{i=1}^n \int_0^\tau K_h(t - t_0) K_b(U_i(t) - u_0) Y_i(t) \frac{\dot{\lambda}_i^*(t, \vartheta_0^*)}{\lambda_i^*(t, \vartheta_0^*)} \Big\{dN_i(t) - \lambda_i^*(t, \vartheta_0^*) dt\Big\} \widetilde{X}_i(t).$$

By the Taylor expansion, we have

$$U_1(\vartheta_0^*|t_0, u_0) = \mathbf{A}_n(t_0, u_0) + \mathbf{B}_n(t_0, u_0) + \mathbf{C}_n(t_0, u_0) + o_p(h^2 + b^2),$$

where

$$\begin{aligned}
&\mathbf{B}_n(t_0, u_0) \\
&= -\frac{1}{2n} \sum_{i=1}^n \int_0^\tau K_h(t - t_0) K_b(U_i(t) - u_0) Y_i(t) \Big[\Big\{\frac{\ddot{\lambda}_{i,0}(t, U_i(t))}{\lambda_{i,0}(t, U_i(t))} - \frac{\dot{\lambda}_{i,0}(t, U_i(t))^2}{\lambda_{i,0}(t, U_i(t))^2}\Big\} \\
&\quad dN_i(t) - \ddot{\lambda}_i^*(t, \vartheta_0^*) dt\Big] \ddot{\alpha}(t_0)^{\mathrm{T}} X_i(t) (t - t_0)^2 \widetilde{X}_i(t), \\
&\mathbf{C}_n(t_0, u_0) \\
&= -\frac{1}{2n} \sum_{i=1}^n \int_0^\tau K_h(t - t_0) K_b(U_i(t) - u_0) Y_i(t) \Big[\Big\{\frac{\ddot{\lambda}_{i,0}(t, U_i(t))}{\lambda_{i,0}(t, U_i(t))} - \frac{\dot{\lambda}_{i,0}(t, U_i(t))^2}{\lambda_{i,0}(t, U_i(t))^2}\Big\} \\
&\quad dN_i(t) - \ddot{\lambda}_i^*(t, \vartheta_0^*) dt\Big] \ddot{\gamma}(u_0)^{\mathrm{T}} W_i(t) I(N_i(t^-) > 0) (U_i(t) - u_0)^2 \widetilde{X}_i(t).
\end{aligned}$$

Following the arguments in Lemma A.1 in Yin et al. (2008), we conclude that

$$\frac{1}{h^2} \mathbf{B}_n(t_0, u_0) \xrightarrow{\mathcal{P}} \frac{1}{2} \mu_2 b_\alpha(t_0, u_0)$$

and

$$\frac{1}{b^2} \mathbf{C}_n(t_0, u_0) \xrightarrow{\mathcal{P}} \frac{1}{2} \mu_2 b_\gamma(t_0, u_0).$$

Therefore, Lemma 2 holds. □

Proof of Theorem 1

Proof of Part (a). By Lemma 1, $\hat{\alpha}(t,u) \xrightarrow{\mathcal{P}} \alpha_0(t)$ uniformly in $t \in [t_1,t_2], u \in [u_1,u_2]$ as $n \to \infty$. Then,

$$\sup_{t\in[t_1,t_2]} |\hat{\alpha}(t) \xrightarrow{\mathcal{P}} \alpha_0(t)| = \sup_{t\in[t_1,t_2]} |n^{-1}\sum_{i=1}^{n}\{\hat{\alpha}(t,U_i(t)) - \alpha_0(t)\}$$
$$\leq \sup_{t\in[t_1,t_2],u\in[u_1,u_2]} |\hat{\alpha}(t,u) - \alpha_0(t)| = o_p(1).$$

□

Proof of Part (b). Under Conditions C.1-C.5, by Lemma 2, we have

$$\sqrt{nh}\{\hat{\alpha}(t_0,u_0) - \alpha_0(t_0) - \frac{1}{2}h^2\mu_2\ddot{\alpha}_0(t_0)\}$$
$$= \mathcal{I}_1 D^{-1}(t_0,u_0)\sqrt{\frac{h}{n}}\sum_{i=1}^{n}\int_0^\tau K_h(t-t_0)K_b(U_i(t)-u_0)Y_i(t)\frac{\dot{\lambda}_{i,0}(t,U_i(t))}{\lambda_{i,0}(t,U_i(t))}\widetilde{X}_i(t)dM_i(t) + o_p(1).$$

Then, by Lemma A.1 in Yin et al. (2008), we obtain

$$\sqrt{nh}\{\hat{\alpha}(t_0) - \alpha_0(t_0) - \frac{1}{2}h^2\mu_2\ddot{\alpha}(t_0)\}$$
$$= \sqrt{\frac{h}{n}}\sum_{i=1}^{n}\int_0^\tau K_h(t-t_0)Y_i(t)\frac{\dot{\lambda}_{i,0}(t,U_i(t))}{\lambda_{i,0}(t,U_i(t))}$$
$$\mathcal{I}_1\Big\{\frac{1}{n}\sum_{j=1}^{n}K_b(U_i(t)-U_j(t_0))D^{-1}(t_0,U_j(t_0))\Big\}\widetilde{X}_i(t)dM_i(t) + o_p(1)$$
$$= \sqrt{nh}\mathbf{A}_n^{(\alpha)}(t_0) + o_p(1),$$

where

$$\mathbf{A}_n^{(\alpha)}(t_0) = \frac{1}{n}\sum_{i=1}^{n}\int_0^\tau K_h(t-t_0)Y_i(t)\frac{\dot{\lambda}_{i,0}(t,U_i(t))}{\lambda_{i,0}(t,U_i(t))}\mathcal{I}_1 D^{-1}(t_0,U_i(t))\widetilde{X}_i(t)dM_i(t).$$

$\sqrt{nh}\mathbf{A}_n^{(\alpha)}(t_0)$ is a sum of local square integrable martingales, with the predictable variation process

$$\Big\langle\sqrt{nh}\mathbf{A}_n^{(\alpha)}, \sqrt{nh}\mathbf{A}_n^{(\alpha)}\Big\rangle(t_0)$$
$$= \frac{h}{n}\sum_{i=1}^{n}\int_0^\tau K_h(t-t_0)^2Y_i(t)\frac{\dot{\lambda}_{i,0}(t,U_i(t))^2}{\lambda_{i,0}(t,U_i(t))}\Big\{\mathcal{I}_1 D^{-1}(t_0,U_i(t))\widetilde{X}_i(t)\Big\}^{\otimes 2}dt$$
$$\xrightarrow{\mathcal{P}} \Sigma_\alpha(t_0),$$

as $n \to \infty$, where

$$\Sigma_\alpha(t_0) = \nu_0 E\Big[Y_i(t_0)\frac{\dot\lambda_{i,0}(t_0, U_i(t_0))^2}{\lambda_{i,0}(t_0, U_i(t_0))}\Big\{\mathcal{I}_1 D^{-1}(t_0, U_i(t_0))\widetilde{X}_i(t_0)\Big\}^{\otimes 2}\Big].$$

Moreover, the Lindeberg type conditions can be easily checked in the following. Let $X_{1,ij}(t, t_0)$ is the jth element of vector $\mathcal{I}_1 D^{-1}(t_0, U_i(t))\widetilde{X}_i(t)$. Then the jth element of $\sqrt{nh}\mathbf{A}_n^{(\alpha)}(t_0)$ is

$$\sqrt{nh}A_{j,n}^{(\alpha)}(t_0) = \frac{h}{n}\sum_{i=1}^{n}\int_0^\tau K_h(t-t_0)Y_i(t)\frac{\dot\lambda_{i,0}(t, U_i(t))}{\lambda_{i,0}(t, U_i(t))}X_{1,ij}(t, t_0)dM_i(t).$$

For any $\epsilon > 0$, let

$$\begin{aligned}\sqrt{nh}A_{j,n,\epsilon}^{(\alpha)}(t_0) = \sqrt{\frac{h}{n}}\sum_{i=1}^{n}\int_0^\tau & K_h(t-t_0)Y_i(t)\frac{\dot\lambda_{i,0}(t, U_i(t))}{\lambda_{i,0}(t, U_i(t))}X_{1,ij}(t, t_0) \\ & I\Big(\sqrt{\frac{h}{n}}\Big|K_h(t-t_0)Y_i(t)\frac{\dot\lambda_{i,0}(t, U_i(t))}{\lambda_{i,0}(t, U_i(t))}X_{1,ij}(t, t_0)\Big| > \epsilon\Big)dM_i(t).\end{aligned}$$

Then the predictable variation process of $\sqrt{nh}A_{j,n,\epsilon}^{(\alpha)}(t_0)$ equals

$$\begin{aligned}&\big\langle\sqrt{nh}A_{j,n,\epsilon}^{(\alpha)}, \sqrt{nh}A_{j,n,\epsilon}^{(\alpha)}\big\rangle(t_0) \\ &= \frac{h}{n}\sum_{i=1}^{n}\int_0^\tau K_h(t-t_0)^2 Y_i(t)\frac{\dot\lambda_{i,0}(t, U_i(t))^2}{\lambda_{i,0}(t, U_i(t))}X_{1,ij}(t, t_0)^2 \\ &\quad I\Big(\sqrt{\frac{h}{n}}\Big|K_h(t-t_0)Y_i(t)\frac{\dot\lambda_{i,0}(t, U_i(t))}{\lambda_{i,0}(t, U_i(t))}X_{1,ij}(t, t_0)\Big| > \epsilon\Big)dt \\ &\xrightarrow{\mathcal{P}} 0,\end{aligned}$$

as $n \to \infty$. By the martingale central limit theorem (cf., Theorem 5.1.1 in Fleming and Harrington (2013)), we conclude that as $n \to \infty$,

$$\sqrt{nh}(\hat\alpha(t) - \alpha_0(t) - \frac{1}{2}h^2\mu_2\ddot\alpha(t)) \xrightarrow{\mathcal{D}} N\left(0, \Sigma_\alpha(t)\right), \text{for } t \in [t_1, t_2].$$

□

Proof of Theorem 2

Proof of Part (a). Similar to the proof of Theorem 1 (a), the consistency of $\hat\gamma(u_0)$ can proved by applying Lemma 1. □

Proof of Part (b). Under Conditions C.1-C.5, by Lemma 2, we have

$$\begin{aligned}
&\sqrt{nb}\{\hat{\gamma}(t_0,u_0)-\gamma_0(u_0)-\frac{1}{2}b^2\mu_2\ddot{\gamma}_0(u_0)\}\\
&=\mathcal{I}_2D^{-1}(t_0,u_0)\sqrt{\frac{b}{n}}\sum_{i=1}^{n}\int_0^{\tau}K_h(t-t_0)K_b(U_i(t)-u_0)Y_i(t)\frac{\dot{\lambda}_{i,0}(t,U_i(t))}{\lambda_{i,0}(t,U_i(t))}\widetilde{X}_i(t)dM_i(t)+o_p(1).
\end{aligned}$$

By Lemma A.1 in Yin et al. (2008), if $n_{u_0}\asymp n$, we obtain

$$\begin{aligned}
&\sqrt{nb}\{\hat{\gamma}(u_0)-\gamma_0(u_0)-\frac{1}{2}b^2\mu_2\ddot{\gamma}(u_0)\}\\
&=\sqrt{\frac{b}{n}}\sum_{i=1}^{n}\int_0^{\tau}K_b(U_i(t)-u_0)Y_i(t)\frac{\dot{\lambda}_{i,0}(t,U_i(t))}{\lambda_{i,0}(t,U_i(t))}\\
&\quad\mathcal{I}_2\Big\{n_{u_0}^{-1}\sum_{t_{u_0}\in\mathcal{V}_{u_0}}K_h(t-t_{u_0})D^{-1}(t_{u_0},u_0)\Big\}\widetilde{X}_i(t)dM_i(t)+o_p(1)\\
&=\sqrt{nb}\mathbf{A}_n^{(\gamma)}(u_0)+o_p(1),
\end{aligned}$$

where

$$\mathbf{A}_n^{(\gamma)}(u_0)=\frac{1}{n}\sum_{i=1}^{n}\int_0^{\tau}K_b(U_i(t)-u_0)Y_i(t)\frac{\dot{\lambda}_{i,0}(t,U_i(t))}{\lambda_{i,0}(t,U_i(t))}\mathcal{I}_2D^{-1}(t,u_0)\widetilde{X}_i(t)dM_i(t).$$

Here $\sqrt{nb}\mathbf{A}_n^{(\gamma)}(u_0)$ is the sum of local square integrable martingales, with the predictable variation process

$$\begin{aligned}
&nb\langle\mathbf{A}_n^{(\gamma)},\mathbf{A}_n^{(\gamma)}\rangle(u_0)\\
&=\frac{b}{n}\sum_{i=1}^{n}\int_0^{\tau}K_b(U_i(t)-u_0)^2Y_i(t)\frac{\dot{\lambda}_{i,0}(t,U_i(t))^2}{\lambda_{i,0}(t,U_i(t))}\Big\{\mathcal{I}_2D^{-1}(t,u_0)\widetilde{X}_i(t)\Big\}^{\otimes 2}dt\\
&\xrightarrow{\mathcal{P}}\Sigma_\gamma(u_0),
\end{aligned}$$

as $n\to\infty$.

Let $X_{2,ij}(t,u_0)$ be the jth element of vector $\mathcal{I}_2D^{-1}(t,u_0)\widetilde{X}_i(t)$. For any $\epsilon>0$, let

$$\begin{aligned}
\sqrt{nb}A_{j,n,\epsilon}^{(\gamma)}(u_0)&=\sqrt{\frac{b}{n}}\sum_{i=1}^{n}\int_0^{\tau}K_b(U_i(t)-u_0)Y_i(t)\frac{\dot{\lambda}_{i,0}(t,U_i(t))}{\lambda_{i,0}(t,U_i(t))}X_{2,ij}(t,t_0)\\
&\qquad I\Big(\sqrt{\frac{b}{n}}\Big|K_b(U_i(t)-u_0)Y_i(t)\frac{\dot{\lambda}_{i,0}(t,U_i(t))}{\lambda_{i,0}(t,U_i(t))}X_{2,ij}(t,t_0)\Big|>\epsilon\Big)dM_i(t).
\end{aligned}$$

Then the predictable variation process of $\sqrt{nb}A^{(\gamma)}_{j,n,\epsilon}(u_0)$ equals

$$
\begin{aligned}
& nb\langle A^{(\gamma)}_{j,n,\epsilon}, A^{(\gamma)}_{j,n,\epsilon}\rangle(u_0) \\
& = \frac{b}{n}\sum_{i=1}^{n}\int_0^{\tau} K_b(U_i(t)-u_0)^2 Y_i(t)\frac{\dot{\lambda}_{i,0}(t,U_i(t))^2}{\lambda_{i,0}(t,U_i(t))} X_{2,ij}(t,u_0)^{\otimes 2} \\
& \quad I\Big(\sqrt{\frac{b}{n}}\Big|K_b(U_i(t)-u_0)Y_i(t)\frac{\dot{\lambda}_{i,0}(t,U_i(t))}{\lambda_{i,0}(t,U_i(t))}X_{2,ij}(t,u_0)\Big| > \epsilon\Big)dt \\
& \xrightarrow{\mathcal{P}} 0,
\end{aligned}
$$

as $n \to \infty$. By the martingale central limit theorem (cf., Theorem 5.1.1 in Fleming and Harrington (2013)), we have as $n \to \infty$,

$$
\sqrt{nb}(\hat{\gamma}(u) - \gamma_0(u) - \frac{1}{2}b^2\mu_2\ddot{\gamma}_0(u)) \xrightarrow{\mathcal{D}} N\left(0, \Sigma_\gamma(u)\right), \text{ for } u \in [u_1, u_2],
$$

where

$$
\Sigma_\gamma(u) = \lim_{n\to\infty} bE\Big[\int_0^{\tau} K_b(U_i(t)-u)^2 Y_i(t)\frac{\dot{\lambda}_{i,0}(t,U_i(t))^2}{\lambda_{i,0}(t,U_i(t))}\Big\{\mathcal{I}_2 D^{-1}(t,u)\widetilde{X}_i(t)\Big\}^{\otimes 2}dt\Big].
$$

□

Web Appendix B: Technical notes on the local log-likelihood function (equation (2) of the main paper)

By Daley and Vere-Jones (2003), the likelihood function for the observed data under model (1) can be constructed as follows:

$$
\mathcal{L}(\alpha,\gamma) = \prod_{0\le t\le\tau}\left[\left\{\prod_{i=1}^{n}[Y_i(t)\lambda_i(t)]^{dN_i(t)}\right\}\left\{1-\sum_{i=1}^{n}Y_i(t)\lambda_i(t)dt\right\}^{1-dN_i(t)}\right],
$$

which simplifies to

$$
\mathcal{L}(\alpha,\gamma) = \left\{\prod_{0\le t\le\tau}\prod_{i=1}^{n}[Y_i(t)\lambda_i(t)]^{dN_i(t)}\right\}\exp\left\{-\sum_{i=1}^{n}\int_0^{\tau}Y_i(t)\lambda_i(t)dt\right\},
$$

where $N_i(t) = \sum_{j=1}^{n_i} I(T_{ij} \leq t)$. Taking logarithms, we obtain the log-likelihood function for the observed data:

$$\ell(\alpha, \gamma) = \sum_{i=1}^{n} \int_0^{\tau} \left[\log\left(Y_i(t)\lambda_i(t)\right) dN_i(t) - Y_i(t)\lambda_i(t)dt\right].$$

Let $\mathcal{N}_{t_0}$ be a neighborhood of t_0 and $\mathcal{N}_{u_0}$ a neighborhood of u_0. For $t \in \mathcal{N}_{t_0}$ and $U_i(t) \in \mathcal{N}_{u_0}$, the intensity $\lambda_i(t) = g^{-1}\{\alpha^T(t)X_i(t) + \gamma^T(U_i(t))Z_i(t)$ can be approximated by

$$\lambda_i^*(t, \vartheta^*|t_0, u_0) = \varphi\{\vartheta^{*T}(t_0, u_0)\tilde{X}_i^*(t, t_0, u_0)\},$$

where $\varphi(\cdot) = g^{-1}(\cdot)$, $\vartheta^*(t_0, u_0) = (\alpha^T(t_0), \gamma^T(u_0), \dot{\alpha}^T(t_0), \dot{\gamma}^T(u_0))^T$, $\tilde{X}_i^*(t|t_0, u_0) = (X_i^T(t), Z_i^T(t), X_i^T(t)(t - t_0), Z_i^T(t)(U_i(t) - u_0))^T$.

Applying the local linear smoothing method (Fan and Gijbels, 1996; Qi et al., 2017) and plugging in the above approximated intensity, the local log-likelihood function for $\alpha(\cdot)$ and $\gamma(\cdot)$ at (t_0, u_0) is:

$$\ell_\vartheta(\vartheta^*|t_0, u_0) = \sum_{i=1}^{n} \int_0^{\tau} K_{h,b}(t, U_i(t)|t_0, u_0)\Big[\log(\lambda_i^*(t, \vartheta^*|t_0, u_0))\, dN_i(t) - Y_i(t)\lambda_i^*(t, \vartheta^*|t_0, u_0)\, dt\Big].$$

Web Appendix C: Additional simulation results

Simulation results for additive models of Section 4 of the main paper

[Web Figure 1 about here.]

[Web Figure 2 about here.]

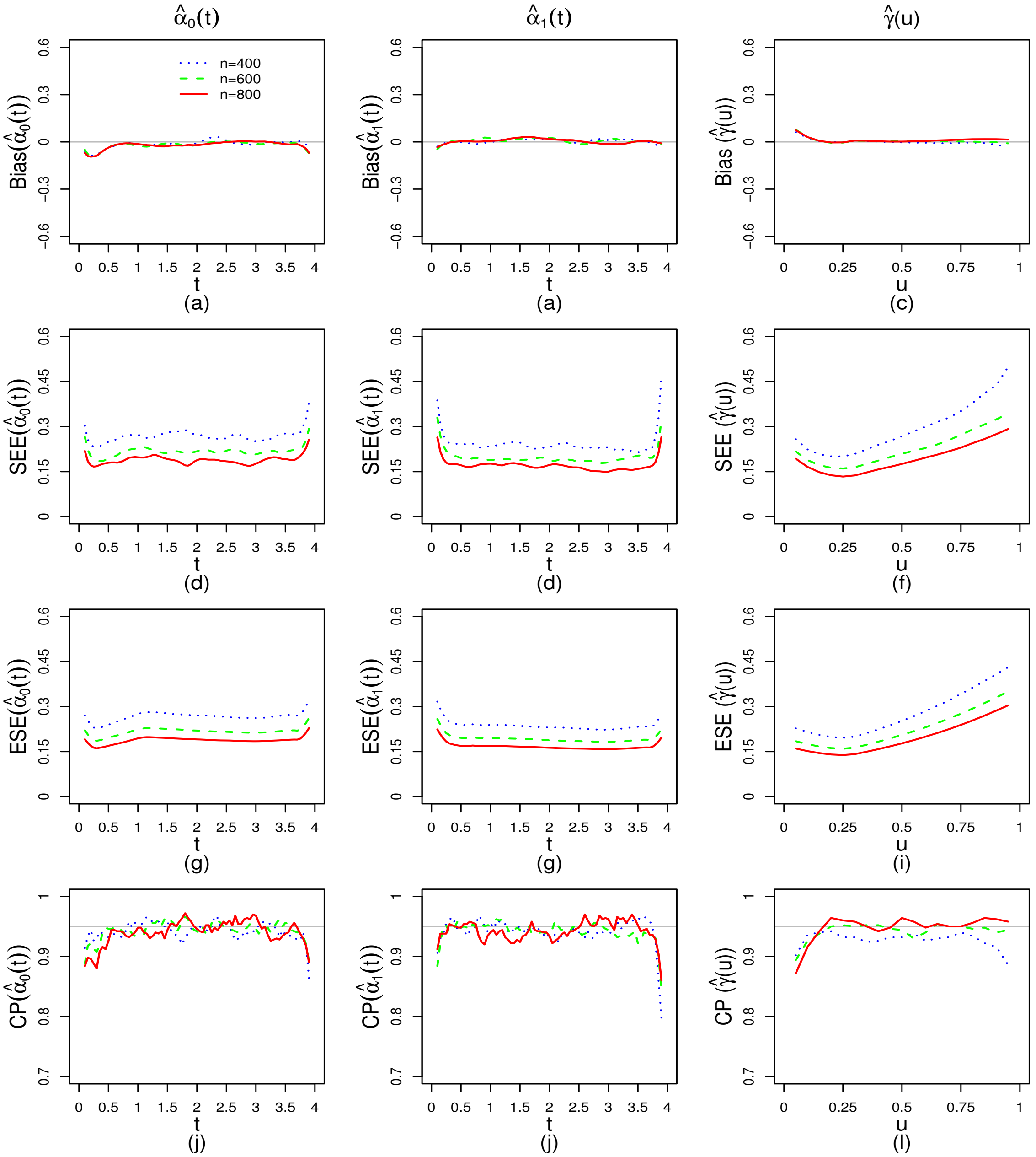

Web Figure 1: Estimation results for $\hat{\alpha}_0(t)$, $\hat{\alpha}_1(t)$ and $\hat{\gamma}(u)$ under model (8). In each panel (left for $\hat{\alpha}_0(t)$, middle for $\hat{\alpha}_1(t)$, and right for $\hat{\gamma}(u)$), lines represent different sample sizes: blue dotted for $n = 400$, green dashed for $n = 600$, and red solid for $n = 800$. The results are based on 500 repetitions. Bias, SEE, ESE, and CP stand, respectively, for the bias, empirical standard error, average estimated standard errors, and 95% empirical coverage probabilities.

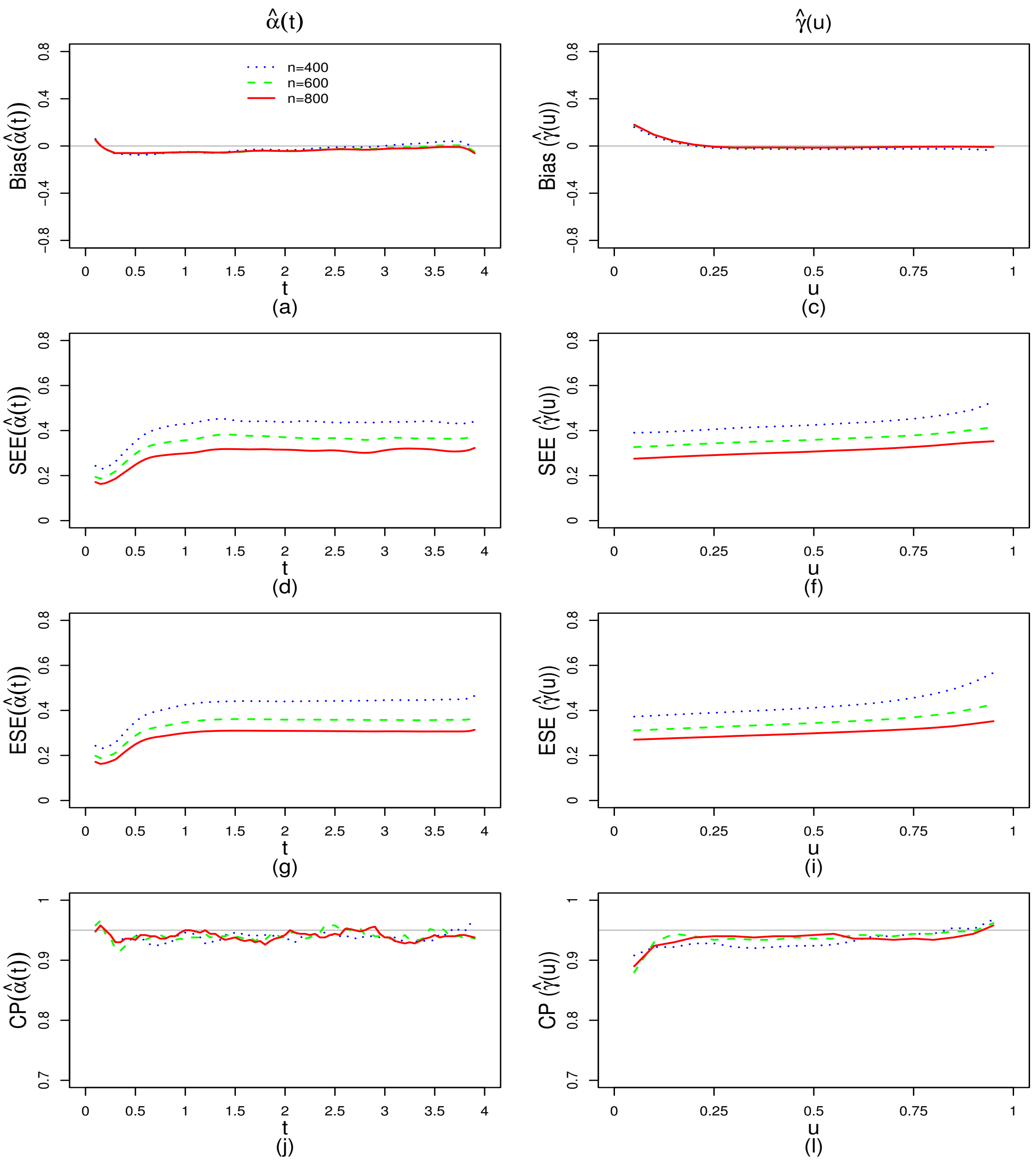

Web Figure 2: Estimation results for $\hat{\alpha}(t)$ and $\hat{\gamma}(u)$ under model (10). In each panel (left for $\hat{\alpha}(t)$ and right for $\hat{\gamma}(u)$), lines represent different sample sizes: blue dotted for $n = 400$, green dashed for $n = 600$, and red solid for $n = 800$. The results are based on 500 repetitions. Bias, SEE, ESE, and CP stand, respectively, for the bias, empirical standard error, average estimated standard errors, and 95% empirical coverage probabilities.

Simulation results with expanded covariates beyond the intercept

To evaluate the performance of the proposed adaptive estimation procedure in a more realistic setting, we consider an extended model where the covariate vectors $X_i(t)$ and $W_i(t)$ include a binary covariate $B_i \sim \text{Bernoulli}(0.5)$. The intensity function is specified as follows:

$$\lambda_i(t) = \exp\left\{\alpha_0(t) + \alpha_1(t)B_i + I(N_i(t^-) > 0)\left[\gamma_0(t - T_{N_i(t^-)}) + \gamma_1(t - T_{N_i(t^-)})B_i\right]\right\}.$$

where

$$\alpha_0(t) = 1.1 - \log(1 + 0.2\log(1 + t)), \quad \alpha_1(t) = -0.4 + 0.05t,$$

and

$$\gamma_0(u) = -\frac{1 - u}{\exp(1 - u)^2}, \quad \gamma_1(u) = -\frac{0.4}{1 + u}.$$

Web Figure 3 presents the simulation results across sample sizes $n = 400$, 600, and 800. The proposed estimator demonstrates excellent performance across all components of $\alpha(t)$ and $\gamma(u)$. These results indicate that our adaptive estimation procedure continues to perform well in this more complex scenario where covariate structures are more complex than intercept-only specifications.

[Web Figure 3 about here.]

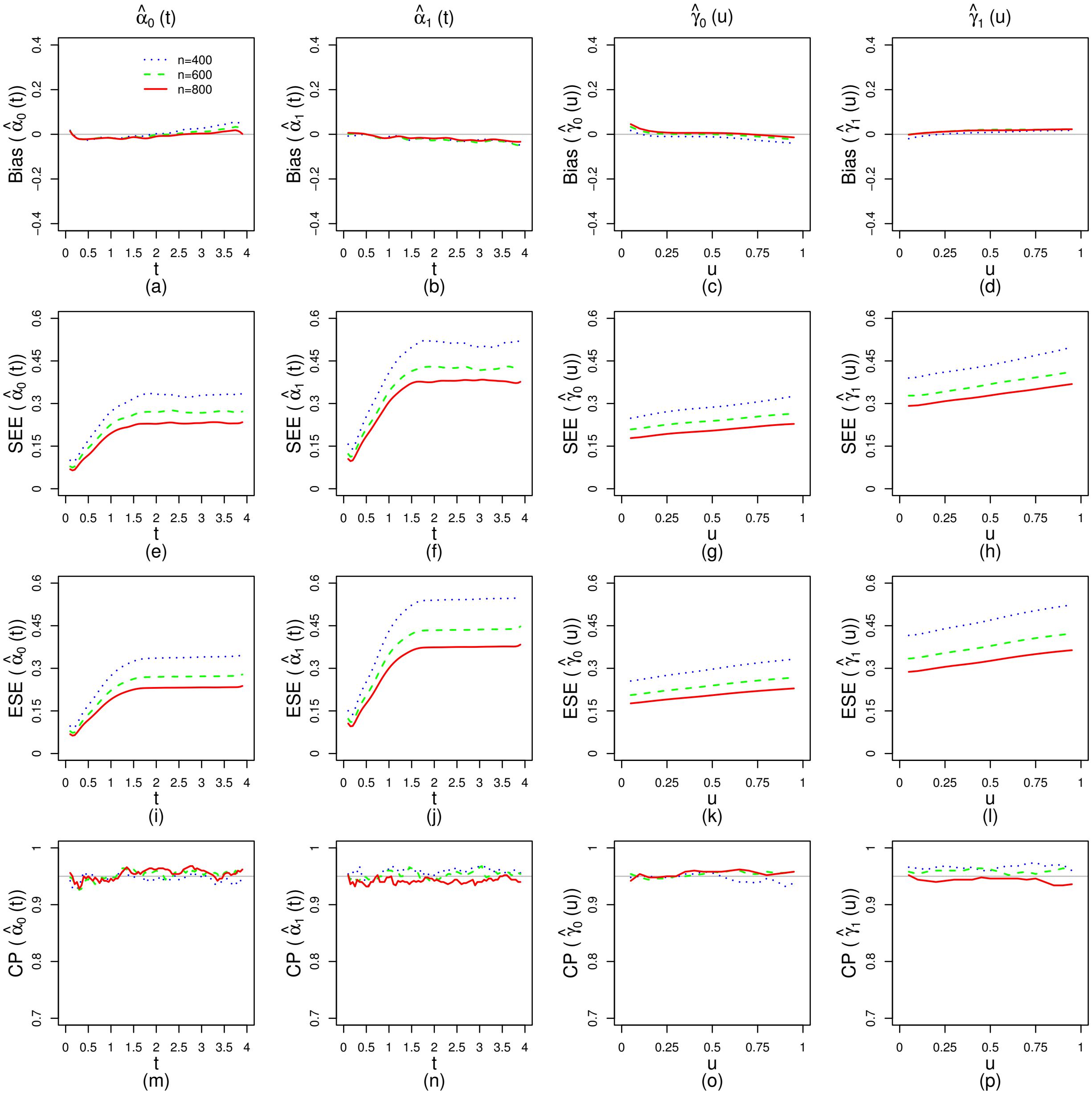

Web Figure 3: Simulation results with $X_i(t) = (1, B_i)$ and $W_i(t) = (1, B_i)$. Each column corresponds to $\alpha_0(t)$, $\alpha_1(t)$, $\gamma_0(u)$, and $\gamma_1(u)$, respectively. Rows show bias, SEE, ESE, and CP for sample sizes $n = 400$, 600, and 800.

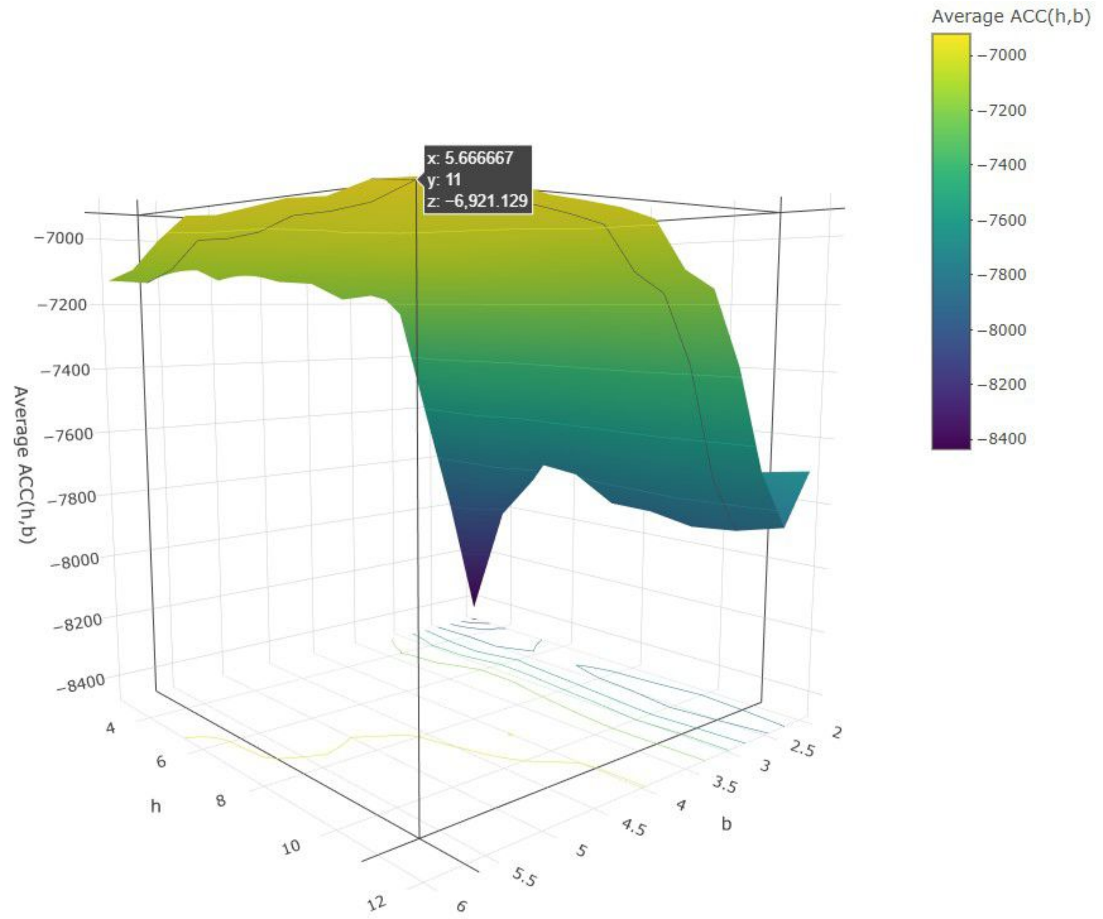

Web Figure 4: A 3D plot for the average $ACC(h, b)$ versus the bandwidth grid points (h, b).

Web Appendix D: Bandwidth selection for the data example

The results of the bandwidth selection process via 5-fold cross-validation method are summarized in the 3D plot presented below. The x and y axes represent the grid points of bandwidths h and b in two dimensions t and u, while the z axis illustrates the corresponding average $ACC(h, b)$ based on ten 5-fold cross-validations defined in Section 2.4 of the main paper. We span h, the bandwidth for t, from 3 months to 12 months with a step of 1 month, and span b, the bandwidth for u, from 2 months to 6 months with a step of 10 days. The optimal bandwidth values which maximized the average $ACC(h, b)$ among all the combinations of these ranges are $h = 11$, $h = 5.667$ (months).

[Web Figure 4 about here.]

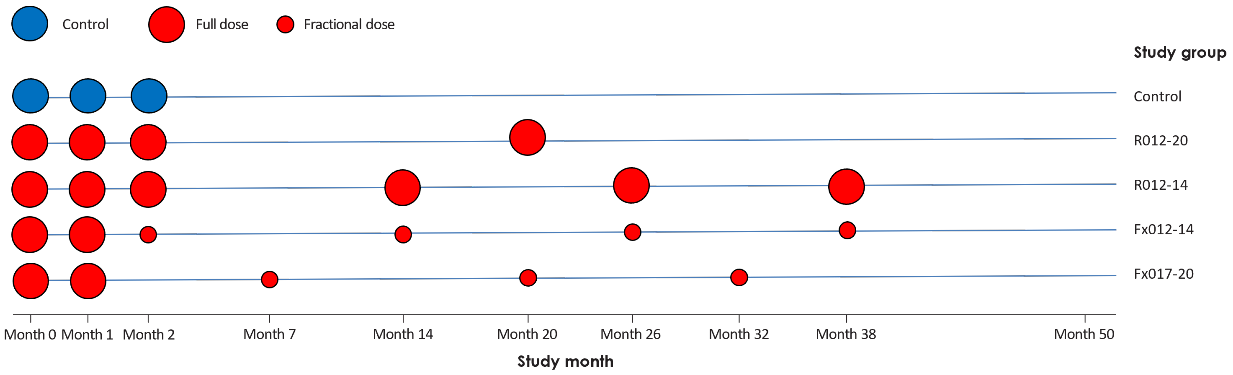

Web Figure 5: Vaccination and dosage schedule for MAL-094 malaria vaccine efficacy trial.

Web Appendix E: Additional analysis of the MAL-094 trial data

In Section 5 of the main paper, we conducted a comprehensive assessment of the dynamic effects associated with the time since the last infection by defining $U_i(t) = t - T_{N_i(t^-)}$. Building on this groundwork, we aim to extend our investigation to ascertain whether vaccine efficacy undergoes variations over time since the last vaccination, particularly exploring whether vaccine efficacy is bolstered by booster doses administered at month 7, month 14, month 20, and month 26 for follow-up through month 32 (Web Figure 5). Such an exploration is essential in providing an understanding of the longevity and adaptive characteristics of vaccine-induced immunity.

[Web Figure 5 about here.]

Let $U_i(t)$ be the time t since the last vaccination, $t - V_{L_i(t^-)}$, where $V_{L_i(t^-)}$ represents the last vaccination time and $L_i(t^-)$ denotes the number of vaccination doses up to time t since enrollment. Two analyses were conducted for the pooled vaccine arm and the control arm separately. For the pooled vaccine arm, the conditional intensity is modeled as

$$\lambda_i^V(t) = \exp\left\{\alpha_0^V(t) + \alpha_1^V(t)\text{Agogo}_i + \alpha_2^V(t)\text{age}_i + \gamma_0^V(t - V_{L_i(t^-)})\right\}. \quad \text{(S.1)}$$

In the estimation process, we set $\gamma_0^V(0) = 0$ to ensure model identification. For the control arm, we consider the following intensity model:

$$\lambda_i^C(t) = \exp\left\{\alpha_0^C(t) + \alpha_1^C(t)\text{Agogo}_i + \alpha_2^C(t)\text{age}_i\right\}. \quad \text{(S.2)}$$

Under model (S.1) and model (S.2), we can define the vaccine efficacy as

$$\text{VE}_i(t) = 1 - \frac{\lambda_i^V(t)}{\lambda_i^C(t)} = 1 - \exp\left\{[\alpha_0^V(t) - \alpha_0^C(t)] + [\alpha_1^V(t) - \alpha_1^C(t)]\text{Agogo}_i + [\alpha_2^V(t) - \alpha_2^C(t)]\text{age}_i + \gamma_0^V(t - V_{L_i(t^-)})\right\}.$$

The results are reported in Web Figure 6 and Web Figure 7. In the first three months, the effectiveness of the vaccine increased as participants in vaccine arms got vaccinated at month 0, month 1, and month 2. Although the analysis was based on pooled vaccine arms, we still can observe that the vaccine efficacy was enhanced due to booster doses at months 7, 14, 20, and 26.

[Web Figure 6 about here.]

[Web Figure 7 about here.]

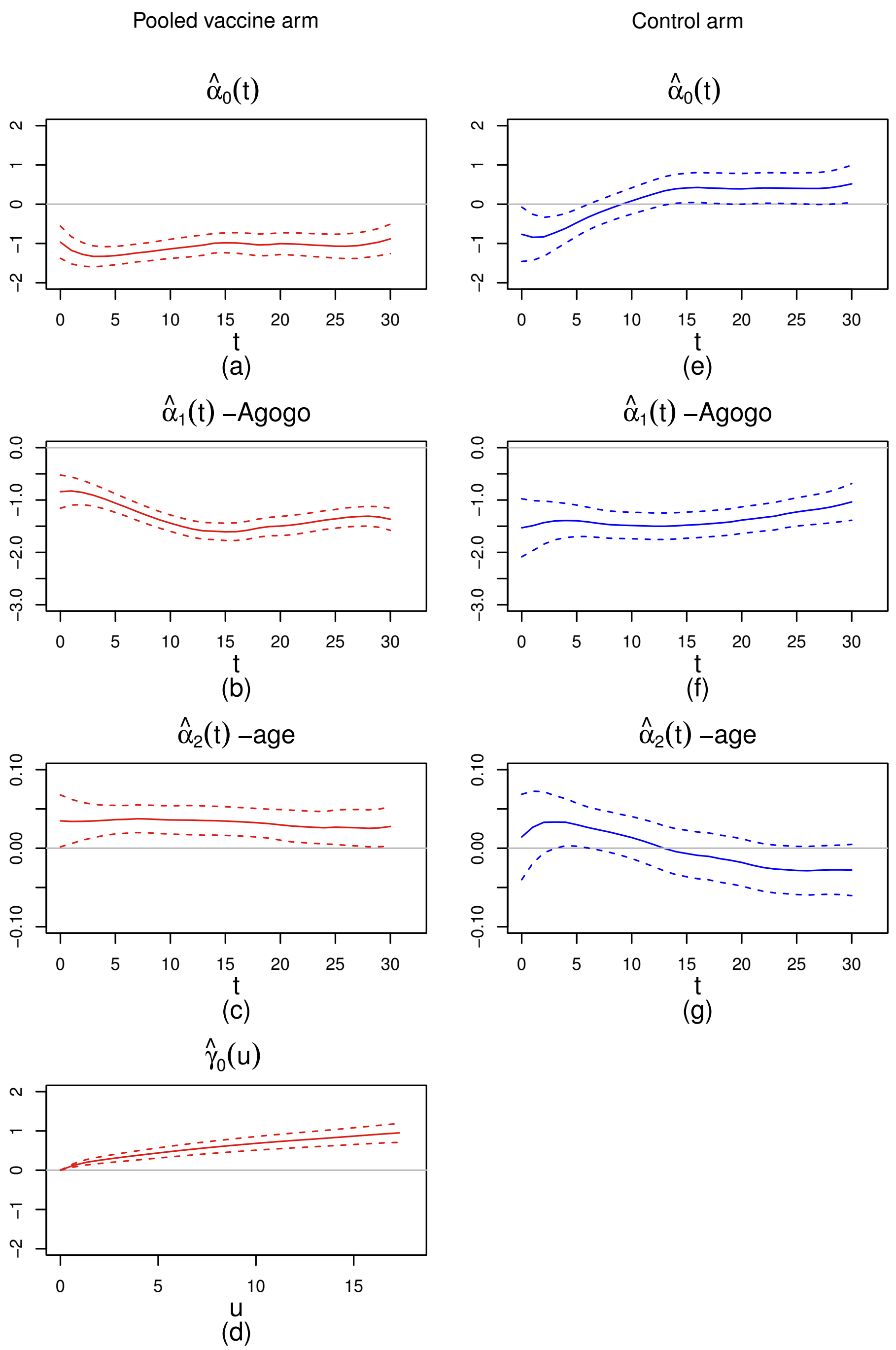

Web Figure 6: Estimation of temporal effects of covariates on malaria infection intensity under models (S.1) and (S.2). The left panels show results for the pooled vaccine arm (red lines), while the right panels show results for the control arm (blue lines). Solid lines represent point estimates, and dashed lines represent the 95% pointwise confidence bands.

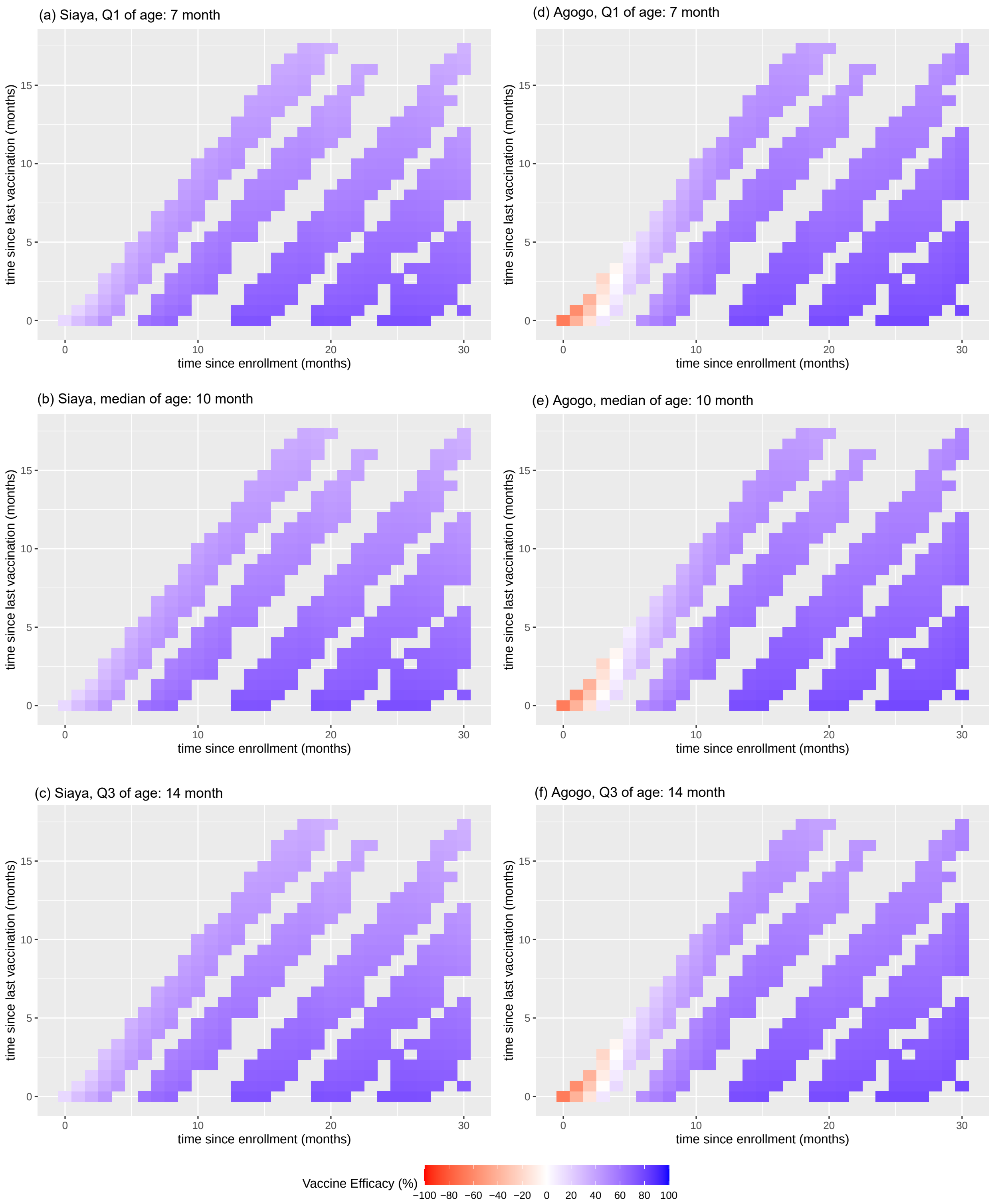

Web Figure 7: Estimation of vaccine efficacy for specific groups under models (S.1) and (S.2).

References

Daley, D. J. and Vere-Jones, D. (2003). *An Introduction to the Theory of Point Processes*. Springer, New York.

Fan, J. and Gijbels, I. (1996). *Local Polynomial Modelling and Its Applications*. Chapman & Hall Ltd, London; New York.

Fleming, T. R. and Harrington, D. P. (2013). *Counting processes and survival analysis*, volume 625. John Wiley & Sons.

Qi, L., Sun, Y., and Gilbert, P. B. (2017). Generalized semiparametric varying-coefficient model for longitudinal data with applications to adaptive treatment randomizations. *Biometrics* **73,** 441–451.

Sun, Y., Wang, H. J., and Gilbert, P. B. (2012). Quantile regression for competing risks data with missing cause of failure. *Statistica Sinica* **22,** 703–728.

Yin, G., Li, H., and Zeng, D. (2008). Partially linear additive hazards regression with varying coefficients. *Journal of the American Statistical Association* **103,** 1200–1213.

Zhang, X., Park, B. U., and Wang, J.-L. (2013). Time-varying additive models for longitudinal data. *Journal of the American Statistical Association* **108,** 983–998.
